# Gut Microbiota Alterations and Their Functional Differences in Depression According to Enterotypes in Asian Individuals

**DOI:** 10.3390/ijms241713329

**Published:** 2023-08-28

**Authors:** Sunmin Park, Chen Li, Xuangao Wu, Tianshun Zhang

**Affiliations:** 1 Department of Food and Nutrition, Obesity/Diabetes Research Center, Hoseo University, Asan-si 31499, Republic of Korea; lic77732@gmail.com (C.L.); tianshunzhang24@gmail.com (T.Z.); 2 Department of Bioconvergence, Hoseo University, Asan-si 31499, Republic of Korea; niyani0@naver.com

**Keywords:** gut microbiota, gut–brain axis, glucose metabolism, network analysis, enterotype

## Abstract

This study aimed to investigate alterations in the gut microbiota of patients with depression compared to those in the gut microbiota of healthy individuals based on enterotypes as a classification framework. Fecal bacteria FASTA/Q samples from 333 Chinese participants, including 107 healthy individuals (Healthy group) and 226 individuals suffering from depression (DP group), were analyzed. The participants were classified into three enterotypes: Bacteroidaceae (ET-B), Lachnospiraceae (ET-L), and Prevotellaceae (ET-P). An α-diversity analysis revealed no significant differences in microbial diversity between the Healthy and DP groups across all enterotypes. However, there were substantial differences in the gut microbial composition for β-diversity, particularly within ET-L and ET-B. The DP group within ET-B exhibited a higher abundance of Proteobacteria, while a linear discriminant analysis (LDA) of the DP group showed an increased relative abundance of specific genera, such as *Mediterraneibacter*, *Blautia*, *Bifidobacterium*, and *Clostridium*. Within ET-L, *Bifidobacterium*, *Blautia*, *Clostridium*, *Collinsella*, and *Corynebacterium* were significantly higher in the DP group in the LDA and ANOVA-like differential expression-2 (ALDEx2) analyses. At the species level of ET-L, *Blautia luti*, *Blautia provencensis*, *Blautia glucerasea*, *Clostridium innocuum*, *Clostridium porci*, and *Clostridium leptum* were the primary bacteria in the DP group identified using the machine learning approach. A network analysis revealed a more tightly interconnected microbial community within ET-L than within ET-B. This suggests a potentially stronger functional relationship among the gut microbiota in ET-L. The metabolic pathways related to glucose metabolism, tryptophan and tyrosine metabolism, neurotransmitter metabolism, and immune-related functions showed strong negative associations with depression, particularly within ET-L. These findings provide insights into the gut–brain axis and its role in the pathogenesis of depression, thus contributing to our understanding of the underlying mechanisms in Asian individuals. Further research is warranted to explain the mechanistic links between gut microbiota and depression and to explore their potential for use in precision medicine interventions.

## 1. Introduction

According to the World Health Organization (WHO), an estimated 5% of adults suffer from depression worldwide, and more women (about 6%) are affected by depression than men (about 4%) [1]. The incidence of depression can vary across populations and regions. In China, depression has increased by about 1.5 fold over the last 30 years and is a significant public health issue [2]. Depression is a complex and heterogeneous psychiatric disorder characterized by persistent sadness, a loss of interest, and a range of emotional and physical symptoms, significantly affecting overall quality of life [3]. However, there are different types of depression, each with specific characteristics and underlying causes, and major depressive disorder, post-stroke depression, and depression with bipolar disorder are forms of depression [4]. Symptoms include changes in appetite and sleep patterns, fatigue, difficulty concentrating, guilt feelings, worthlessness feelings, and recurrent thoughts of death or suicide [3]. The etiology of depression is linked not only to psychosocial factors but also to biological factors. The various factors contributing to depression include genetic predisposition, imbalances in brain chemistry, hormonal changes, life events, chronic medical conditions, and certain medications [5]. Treatment for depression often involves a combination of psychotherapy, medication, lifestyle modifications, and support from healthcare professionals [3,5].

The role of the gut–brain axis, the bidirectional communication between the gut, its microbiome, the nervous system, and food intake, has gained significant attention [6]. In the Rotterdam Study (*n* = 1054) and the Amsterdam Healthy Life in an Urban Setting (HELIUS) cohorts (*n* = 1539), participants with depression had a relative abundance of specific microbial taxa, including *Eggerthella*, *Subdoligranulum*, *Coprococcus*, *Sellimonas*, *Lachnoclostridium*, *Hungatella*, Ruminococcaceae, Lachnospiraceae UCG001, *Eubacterium ventriosum*, and *Ruminococcus gauvreauii* [7]. The gut microbiota of adults with depression appears to be involved in synthesizing key neurotransmitters, namely, glutamate, butyrate, serotonin, and γ-aminobutyric acid (GABA) [7]. They may also be linked to inflammation, immune activation, intestinal permeability, metabolite production, and hypothalamic–pituitary–adrenal (HPA) axis dysregulation. Interventions such as probiotics, prebiotics, and dietary modifications have been explored as strategies to restore healthy gut microbiota and improve the symptoms of depression [8]. However, the field is still evolving, and more studies are needed to elucidate the mechanisms underlying the gut–brain axis.

Long-term dietary patterns significantly influence the composition of the gut microbiota. The gut microbiota has been grouped based on the stable clusters of bacterial communities that co-exist and are distinguished by the dominant bacterial community. The enterotypes are linked to the host’s genetics and dietary components and may correlate with individual health status [9]. The enterotypes reflect distinct microbial compositions and functional profiles within the gut microbiota, and certain enterotypes have been associated with various disease conditions, including depression [10]. The Bacteroides enterotype tends towards lower overall diversity and has been linked to colorectal cancer, non-alcoholic fatty liver, celiac disease, immune senescence, and low-grade inflammation [11]. However, it remains controversial. Prevotella enterotypes might also be linked to metabolic disturbances, insulin resistance, and inflammation, especially in Asian individuals [12,13]. Investigating the prevalence and impact of specific enterotypes in individuals with depression can enhance our understanding of the gut–brain axis and potentially identify the microbial signatures associated with the risk of depression, its severity, or treatment response [14]. Enterotypes provide a framework for initial investigations, but more comprehensive analyses of individual microbial species, functional profiles [11], and their interactions are needed to support a better understanding of the gut–brain connection in depression. Therefore, this study aimed to investigate alterations in the gut microbiota of patients with depression compared to those in the gut microbiota of healthy adults, based on enterotypes, using combined gut microbiota data from previous human studies on Chinese participants. The results are expected to enhance our understanding of the gut–brain axis and its role in the pathogenesis of depression and provide insights for potential diagnostic and therapeutic strategies.

## 2. Results

### 2.1. Collection of Fecal Bacteria and Enterotypes of the Participants

**Figure 1:**
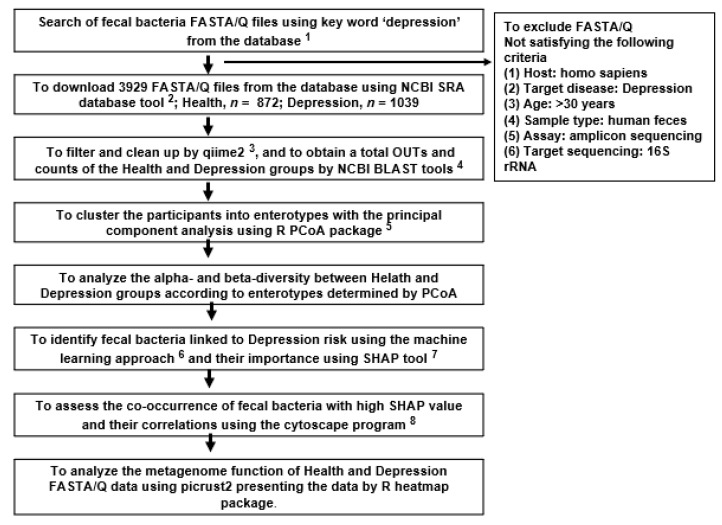
A flowchart of the overall selection process of the critical gut bacteria from fecal FASTA/Q collected from the open database. ^1^ SRA accession list on NCBI SRA database (https://www.ncbi.nlm.nih.gov/sra (accessed on 2 November 2022)) and GMrepo database (https://gmrepo.humangut.info/ (accessed on 6 November 2022)); ^2^ https://trace.ncbi.nlm.nih.gov/Traces/sra/sra.cgi?view=software (accessed on 1 December 2022); ^3^ https://view.qiime2.org/ (accessed on 25 November 2022); ^4^ https://blast.ncbi.nlm.nih.gov/Blast.cgi (accessed on 6 December 2022); ^5^ https://cran.r-project.org/web/packages/aPCoA/index.html (accessed on 5 January 2023); ^6^ https://xgboost.readthedocs.io/en/stable/install.html (accessed on 7 February 2023); ^7^ https://shap.readthedocs.io/en/latest/index.html (accessed on 30 February 2023); ^8^ https://cytoscape.org (accessed on 16 March 2023).

A total of 333 fecal bacteria FASTA/Q files comprising 107 healthy participants in the Healthy group and 226 individuals with depression in the DP group were collected according to the procedure shown in Figure 1. Table S1 presents the project-provided FASTA/Q files used in the present study. They were clustered into three enterotypes satisfying eigenvalue >1.5 via a principal component analysis (PCA): high Bacteroidaceae (ET-B; Healthy: 45, DP: 84), high Lachnospiraceae (ET-L; Healthy: 47, DP: 127), and high Prevotellaceae (ET-P; Healthy: 15, DP: 15) enterotypes. ET-B contained 44.0% Bacteroidaceae, 12.6% Lachnospiraceae, and 1.6% Prevotellaceae; ET-L included 10.1% Bacteroidaceae, 31.1% Lachnospiraceae, and 1.5% Prevotellaceae; ET-P comprised 9.3% Bacteroidaceae, 9.0% Lachnospiraceae, and 53.6% Prevotellaceae (Supplementary Figure S1).

The Chao1, Shannon, and Simpson indices representing α-diversity did not significantly vary between the Healthy and DP groups comprising all the participants and each enterotype (Supplementary Figure S2). The β-diversity used to determine and assess the differences in microbial communities between the Healthy and DP groups, measured using the Bray–Curtis dissimilarity matrix, was significantly different in the participants taken as a whole (*p* < 0.001), ET-L (*p* < 0.001), and ET-B (*p* = 0.006). However, ET-P had no significant β-diversity (Supplementary Figure S3). The β-diversity results suggested that the diversity seen among the gut microbiota of all participants was primarily from ET-L.

### 2.2. Gut Microbiota Composition of Participants with and without Depression in ET-B

**Figure 2:**
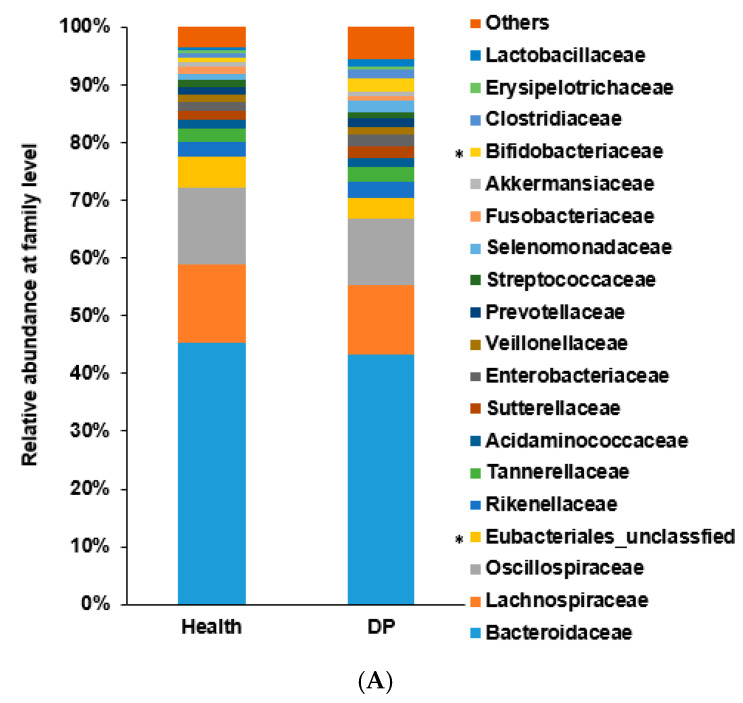

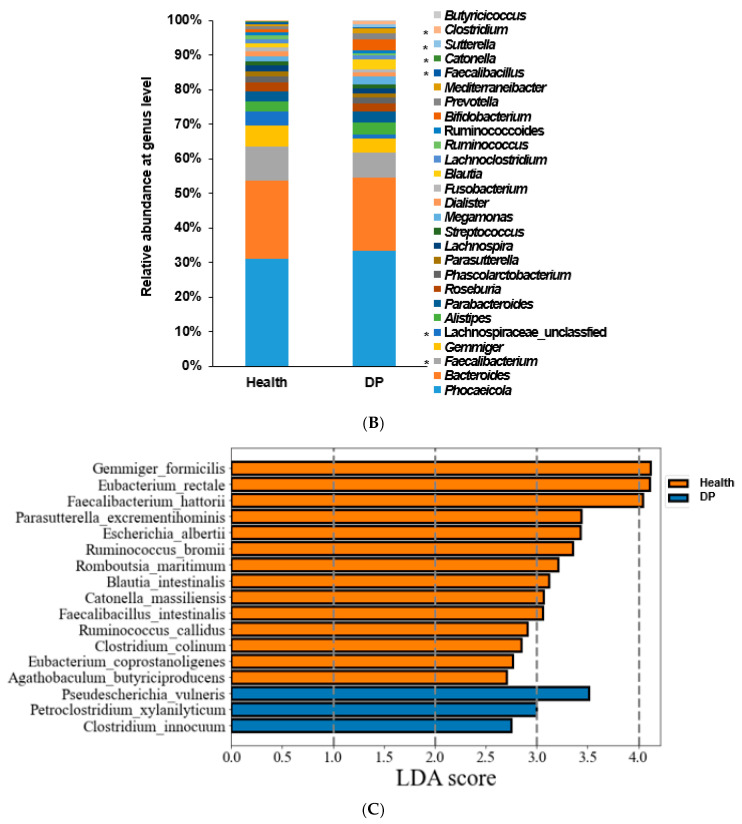

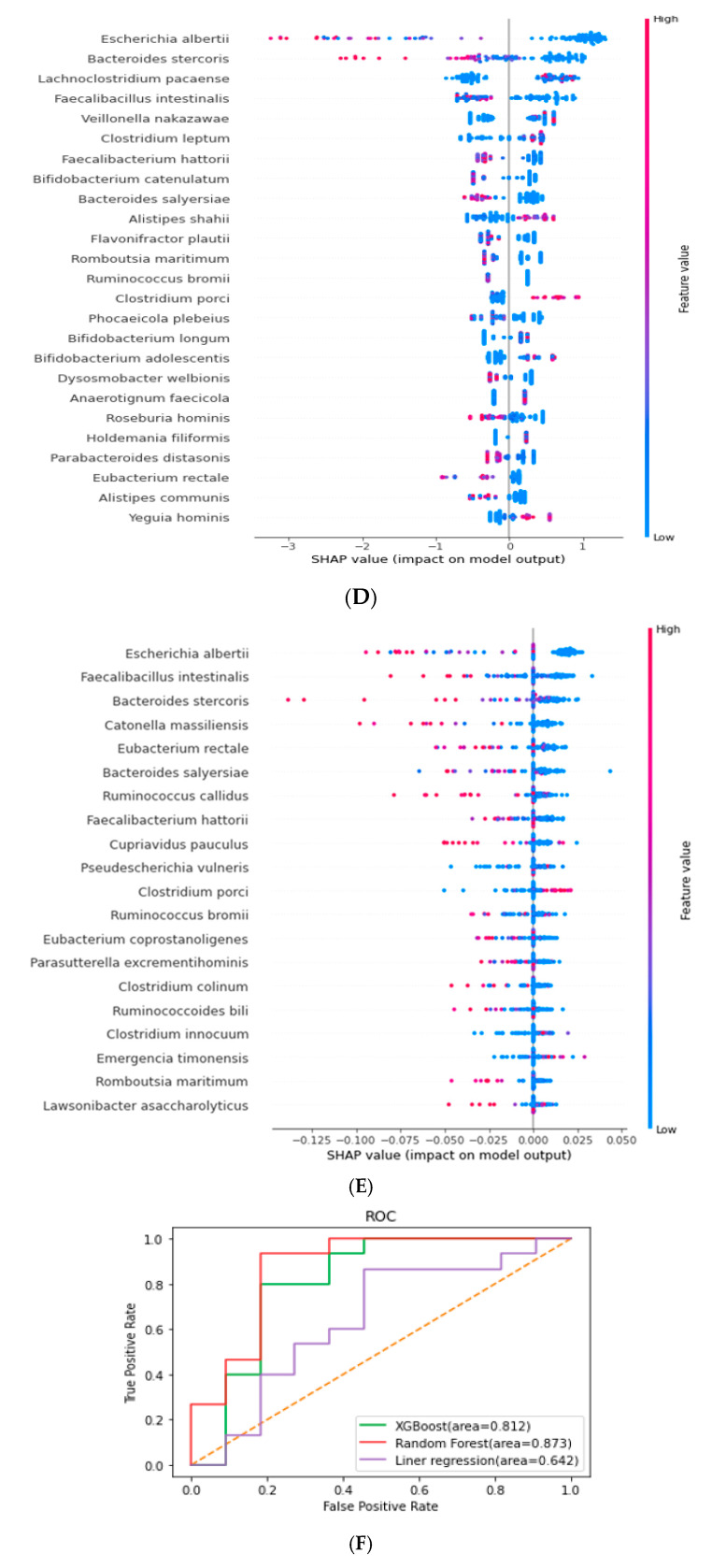
Comparison of the gut microbiota composition between the Healthy and Depressed (DP) groups in ET-B. (**A**) Relative abundance of gut microbiota at the family level. (**B**) Relative abundance of gut microbiota at the genus level. (**C**) Primary gut microbiota in Healthy and DP groups in ET-B using linear discriminant analysis (LDA) scores. (**D**) Primary gut microbiota in Healthy and DP groups at the species level using the XGBoost algorithm. (**E**) Primary gut microbiota in Healthy and DP groups at the species level using the random forest algorithm. (**F**) Area under the curve of ROC. * Significant differences between the DP and Healthy groups at *p* < 0.00001 (Bonferroni corrected *p* value).

At the phylum level in ET-B, the relative abundance of Proteobacteria was higher in the DP group than in the Healthy group (*p* = 0.01). At the family level, the DP group exhibited a higher relative abundance of *Clostridiaceae*, *Odoribacteraceae*, *Eggerthellaceae*, and *Campylobacteraceae* than the Healthy group (*p* < 0.05; Figure 2A). At the genus level, the relative abundance of *Faecalibacterium*, *Faecalibacillus*, and unclassified Lachnospiraceae was higher and the relative abundance of *Clostridium*, *Eggerthella*, *and Mediterraneibacter* was lower in the Healthy group than in the DP group (Figure 2B).

At the species level, the linear discriminant analysis (LDA) effect size (LEfSe) analysis identified *Petroclostridium xylanilyticum*, *Pseudescherichia vulneris*, and *Clostridium innocuum* as having high LDA scores in the DP group (Figure 2C). There was a separation of bacteria at the genus level as presented in Supplementary Figure S4. However, the ANOVA-like differential expression (ALDEx2) analysis did not reveal significant differences in the gut microbiota composition between the Healthy and DP groups at the genus and species levels within the ET-B enterotype.

The primary bacteria for the Healthy and DP groups were selected using machine learning algorithms, such as XGBoost, random forest, and linear regression. In ET-B, XGBoost revealed that *Escherichia albertii*, *Bacteroides stercoris*, *Facalibacterium hattori*, *Roseburia hominis*, *Parabacteroides distasonis*, and *Eubacterium rectale* were higher in the Healthy group than in the DP group, while *Alistipes shahii*, *Clostridium porci*, *Clostridium leptum*, *Clostridium innocuum*, and *Bifidobacterium adolescent* were higher in the DP group (Figure 2D). The random forest algorithm showed that *Clostridium porci* and *Emergencia timonensis* were higher in the DP group. Overall, the findings suggest that specific bacterial species, such as *Clostridium porci*, *Clostridium leptum*, and *Clostridium innocuum*, may play a role in distinguishing the DP group from the Healthy group (Figure 2E). These results indicate that the microbiota in the Healthy and DP groups within ET-B are not distinct and separate. Since the primary bacteria identified through different methods were varied, a clear differentiation of the microbiota between the two groups could not be made. The receiver operating characteristic (AUROC) of the XGboost and random forest indicated that the random forest model might be a better prediction model for depression-related gut bacteria than the XGBoost model (Figure 2F). The accuracy, specificity, sensitivity, and precision values of the models are provided in Supplementary Table S2. They indicated that the prediction models were appropriate to use, but the specificity of the models was low.

### 2.3. Gut Microbiota Composition of Patients with Depression in ET-L

**Figure 3:**
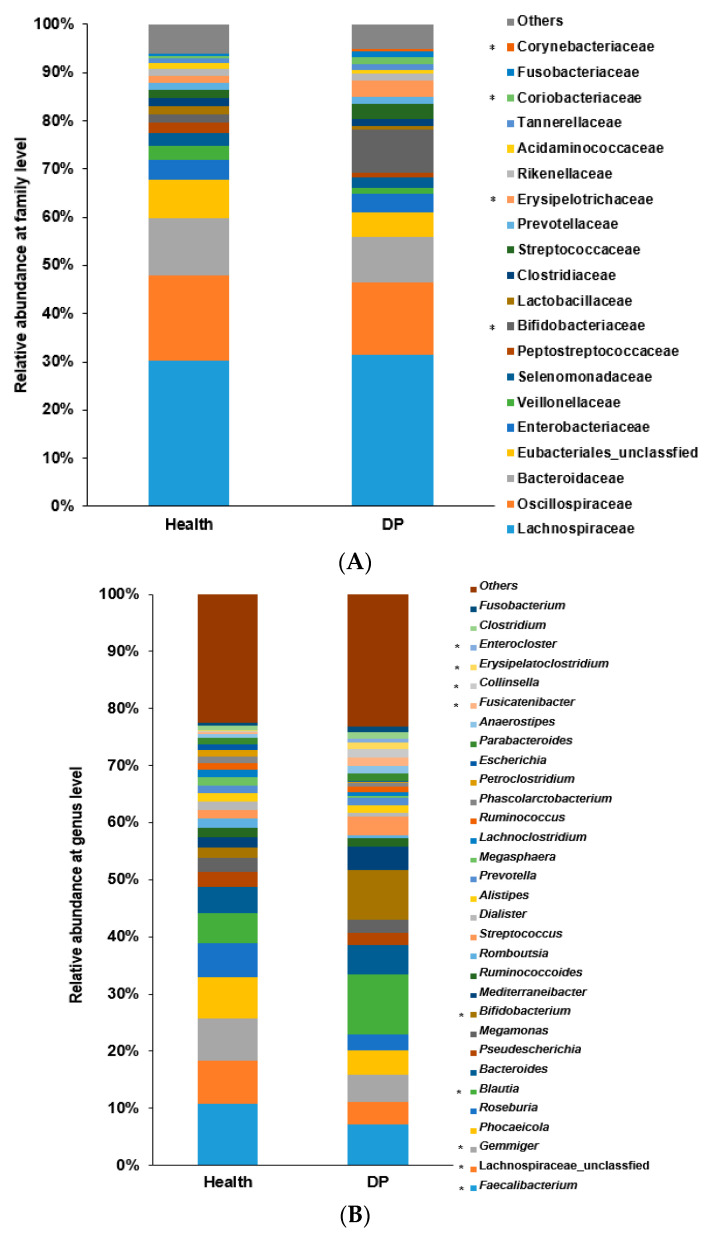

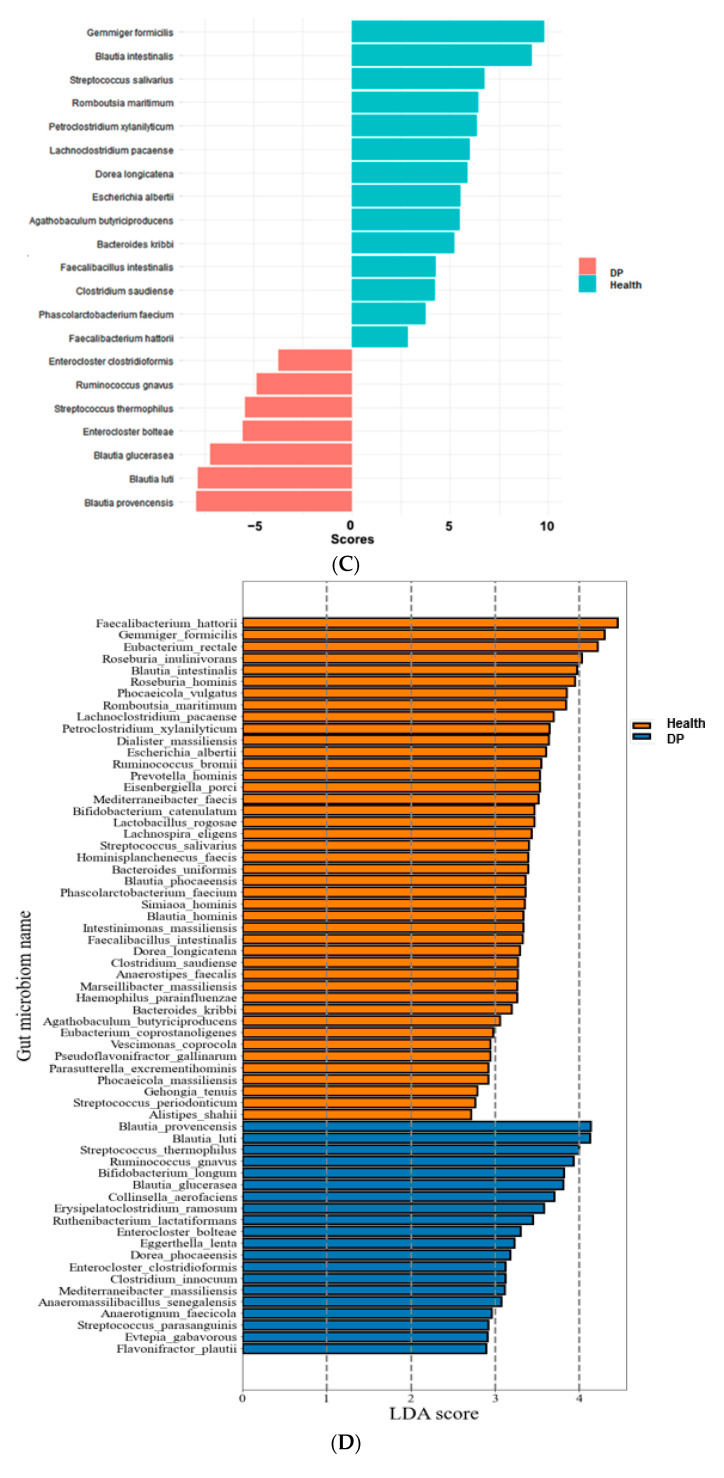

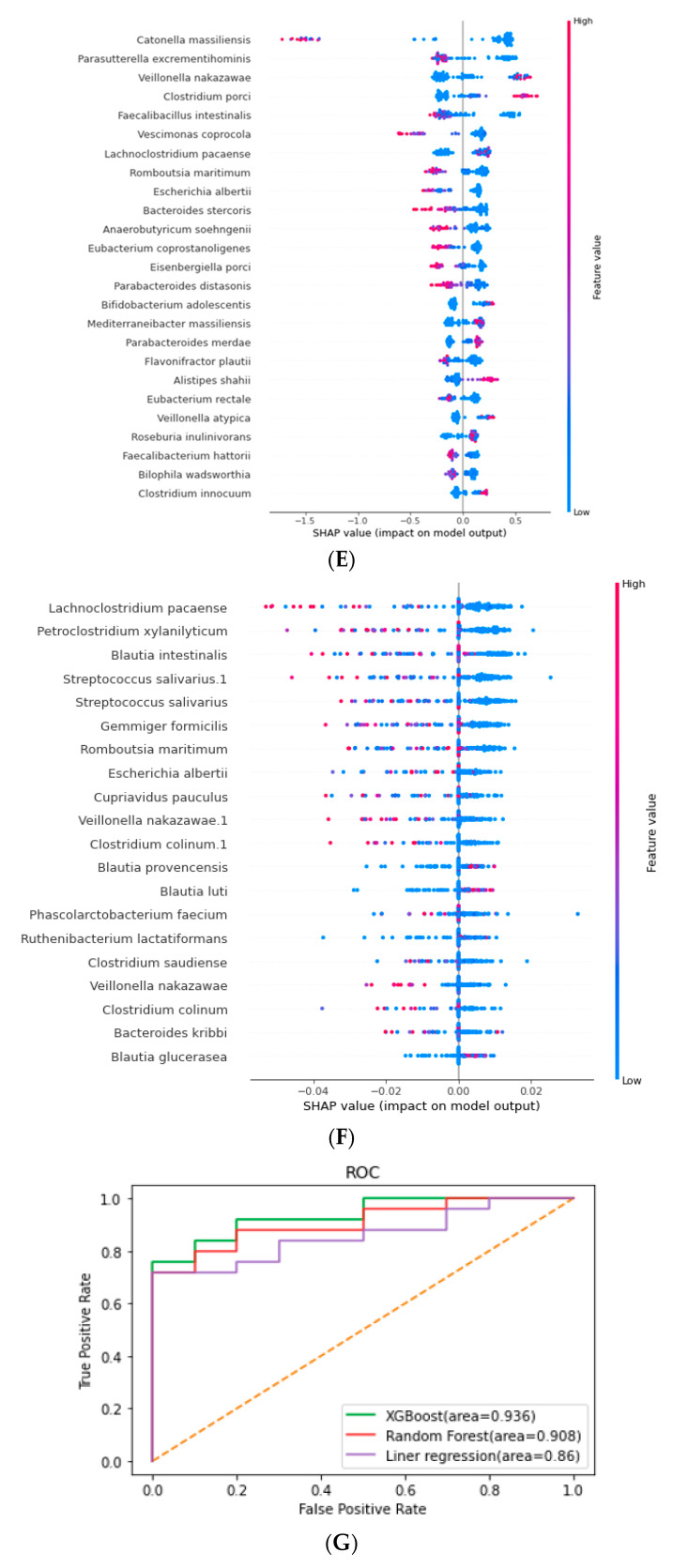
Comparison of the gut microbiota composition between the Healthy and Depressed (DP) groups in ET-L. (**A**) Relative abundance of gut microbiota at the family level. (**B**) Relative abundance of gut microbiota at the genus level. (**C**) Primary gut microbiota in Healthy and DP groups at the species level in ET-B using ALDEx2. (**D**) Primary gut microbiota in Healthy and DP groups at the species level in ET-B using linear discriminant analysis (LDA) scores. (**E**) Primary gut microbiota in Healthy and DP groups at the species level using the XGBoost algorithm. (**F**) Primary gut microbiota in Healthy and DP groups at the species level using the random forest algorithm. (**G**) Area under the curve of ROC. * Significant differences between the DP and Healthy groups at *p* < 0.00001 (Bonferroni corrected *p* value).

At the phylum level in ET-L, the relative abundance of Actinobacteria was significantly higher in the DP group than in the Healthy group (*p* = 3.1 × 10^−11^), while the relative abundance of Proteobacteria did not show significant differences as observed in ET-B. At the family level, the DP group exhibited a higher relative abundance of *Bifidobacteriaceae* (*p* = 1.05 × 10^−8^), *Erysipelotrichaceae* (*p* = 0.0009), *Coriobacteriaceae* (*p* = 1.26 × 10^−7^), and *Corynebacteriaceae* (*p* = 6.07 × 10^−5^) than the Healthy group (Figure 3A). At the genus level, *Bifidobacterium*, *Blautia*, *Collinsella*, *Corynebacterium*, *Faecalibacterium, Enterocloster*, *Erysipelatoclostridium*, *Germiger*, *Lawsonibacter*, and *Fusicatenibacter* were significantly more abundant in the DP group than in the Healthy group, as determined using Bonferroni correction (*p* < 5 × 10^−5^; Figure 3B).

The bacteria were separated at the genus level; the results are presented in Supplementary Figure S5. The ALDEx2 analysis at the species level revealed *Blautia glucerasea*, *Blautia luti*, *Blautia provencensis*, *Enterocloster bolteae*, *Enterocloster clostridioformis*, and *Ruminococcus gnavus* to be associated with the DP group, while *Gemmiger formicilis*, *Blautia intestinalis*, *Dorea longicatena*, *Escherichia albertii*, *Lachnoclostridium pacaense*, *Faecalibacillus intestinalis*, *Faecalibacterium hattori*, and *Bacteroides kribbi* were associated with the Healthy group (Figure 3C). The LDA scores further highlighted the specific bacteria associated with the Healthy and DP groups, with more pronounced differences compared to the ALDEx2 analysis (Figure 3D).

In the machine learning approach, the XGBoost algorithm revealed that *Veillonella nakazawae*, *Clostridium porci*, *Alistipes shahii*, *Veillonella atypica*, and *Clostridium innocuum* were the primary gut microbiota in the DP group (Figure 3E). Meanwhile, the random forest algorithm showed that *Blautia luti*, *Blautia provencensis*, and *Blautia glucerasea* were the major gut microbiota in the DP group (Figure 3F). The XGBoost and random forest algorithms revealed different primary bacteria for the DP group, and the bacteria selected by the algorithms were also part of the ALDEx2 and LDA results. These results suggest that a random forest model could be used to identify the primary gut microbiota for the DP group. Unlike in ET-B, in ET-L, the primary gut microbiota in the DP group was consistent across the different methods used. The gut microbiota was found to be distinct and separate between the Healthy and DP groups in ET-L. The AUROC of the XGboost and random forest indicated that the XGBoost (AUROC = 0.936) and random forest (AUROC = 0.908) models were suitable for predicting depression-related gut bacteria (Figure 3G). The accuracy, specificity, sensitivity, and precision values of the models indicated that they were appropriate for use as prediction models (Table S2).

### 2.4. Gut Microbiota Composition of Patients with Depression in ET-P

There were no significant bacteria at the phylum level in ET-P. The relative abundance of *Erysipelotrichaceae* was higher in the DP group than in the Healthy group (*p* = 0.037) at the family level (Supplementary Figure S4A). At the genus level, the relative abundance of *Clostridium* and *Holdemanella* was higher in the DP group than in the Healthy group (*p* = 0.01; Supplementary Figure S4B). These results suggest no significant bacteria as determined by Bonferroni correction. This could be due to the small sample size. Comparisons of the gut microbiota composition, Aldex2 values, LDA scores, and prediction models between depressed and healthy adults in ET-P are given in Supplementary Figure S5.

### 2.5. Gut Microbiota Interaction Network in ET-B

**Figure 4:**
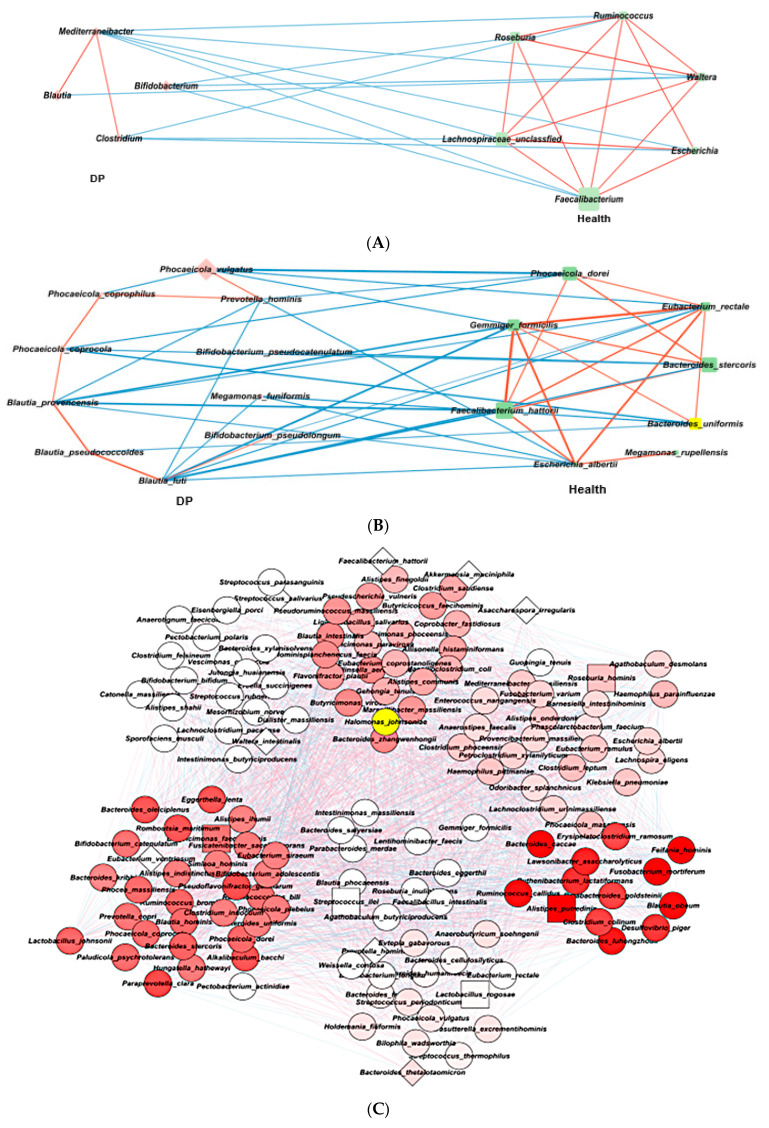
Network of primary gut microbiota in the Healthy and Depressed (DP) groups in ET-B. (**A**) Comparison between the Healthy and Depressed (DP) groups at the genus level. (**B**) Comparison between the Healthy and Depressed (DP) groups at the species level. (**C**) Clusters of gut microbiota with Minimal Common Oncology Data Elements (MCODE). O: clustered bacteria; □: seed bacteria; ◊: unclustered bacteria. The darker red color of nodes indicated higher absolute value of correlation coefficients. The pink and blue lines indicated positive and negative association between the nodes.

Fewer bacteria in ET-B were associated with depression risk, although some bacterial taxa were shared with ET-L (Figure 4A). At the genus level, *Mediterraneibacter, Blautia, Bifidobacterium*, *and Clostridium* were found to be higher in abundance in the DP group. Meanwhile, *Ruminococcus*, *Roseburia*, *Escherichia*, *Faecalibacterium*, and unclassified Lachnospiraceae were higher in the Healthy group.

At the species level, *Bacteroides stercoris*, *Bacteroides uniformis*, *Phocaeicola dorei*, *Faecalibacterium hattori*, *Gemmiger formicilis*, *Escherichia albertii*, and *Megamonas rupeliensis* were found to be higher in abundance in the Healthy group. Conversely, *Bifidobacterium pseudolongum*, *Bifidobacterium pseudocatenulatum*, *Blautia luti*, *Blautia pseudococcoides*, *Blautia provencensis*, *Megamonas funiformis*, *Prevotella hominis*, *Phocaeicola vulgatus*, *Phocaeicola coprophilus*, and *Phocaeicola coprocola* were higher in abundance in the DP group (Figure 4B).

The gut microbiota was clustered into five clusters using an MCODA analysis with a 0.2 node score cutoff, 3 K-core, 4-degree cutoff, and 100 maximum depth. The primary cluster included 11 bacteria, with *Allistipes putredin* is as seed bacteria (Table 1). They had more positive interactions among bacteria than negative ones, and their interactions among bacteria were relatively weak. The microbial interconnections and stability of the gut microbiota in ET-B are presented in Table 2. The microbial interconnections of the gut microbiota network, determined by the average degree and path length, were lower in the DP group than in the Healthy group. They showed six clusters in network analysis of ET-B (Figure S6A). Their stability, determined by the negative edge ratio, was higher in the Healthy group than in the DP group (Figure 4C). This suggests that the network of the gut microbiota in the Healthy group was more complex and stable. Therefore, it would be difficult to alter the microbiota of the Healthy group to that of the DP group in ET-B through any external interventions.

### 2.6. Gut Microbiota Interaction Network in ET-L

**Figure 5:**
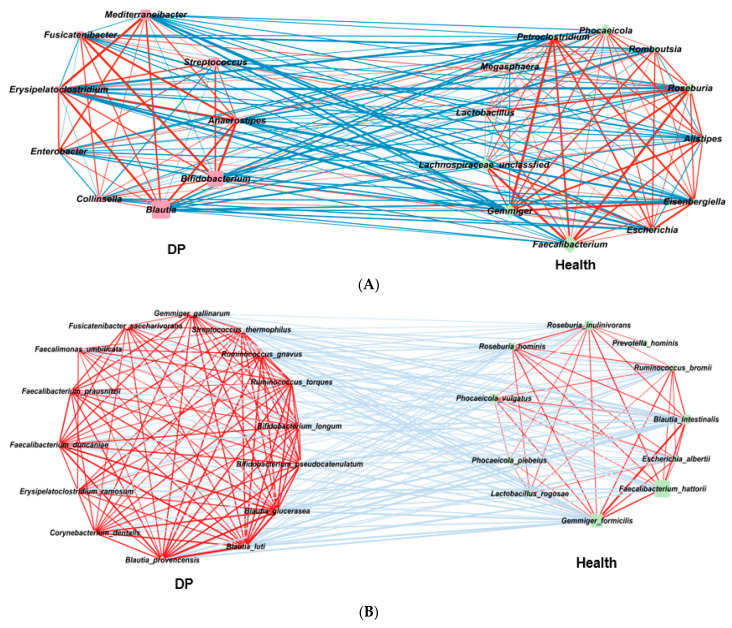

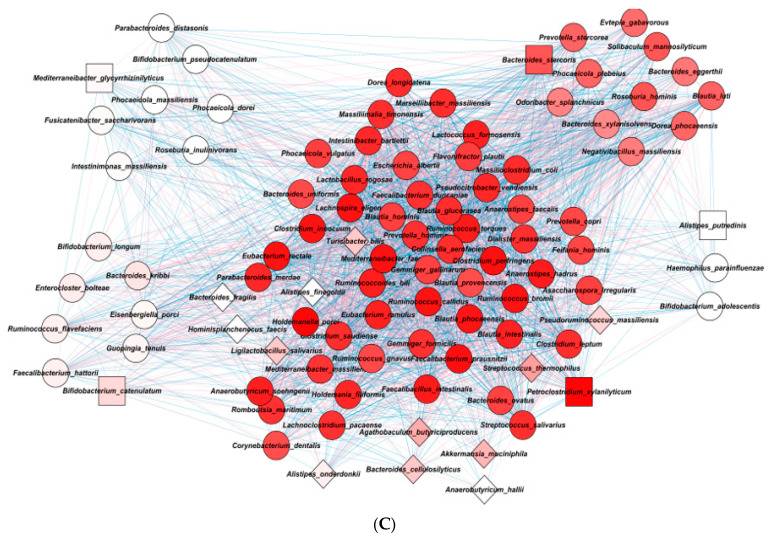
Network of gut microbiota in the Healthy and Depressed (DP) groups in ET-L. (**A**) Comparison between the Healthy and Depressed (DP) groups at the genus level. (**B**) Comparison between the Healthy and Depressed (DP) groups at the species level. (**C**) Clusters of gut microbiota with Minimal Common Oncology Data Elements (MCODE): O: clustered bacteria; □: seed bacteria; ◊: unclustered bacteria. The darker red color of nodes indicated higher absolute value of correlation coefficients. The pink and blue lines indicated positive and negative association between the nodes.

Compared to ET-L, the DP group exhibited a higher abundance of specific bacterial genera at the genus level. Specifically, *Mediterraneibacter*, *Streptococcus*, *Fusicatenibacter*, *Erysipelatoclostridium*, *Clostridium*, *Collinsella*, *Blautia*, *Bifidobacterium*, and *Anaerostipes* were found to be more prevalent in the DP group (Figure 5A). Conversely, the Healthy group showed a higher abundance of *Faecalibacterium*, *Gemmiger*, *Escherichia*, *Allistipes*, *Lactobacillus*, *Roseburia*, *Phocaeicola*, *Megasphaera*, and unclassified Lachnospiraceae (Figure 5A).

At the species level, the specific bacteria associated with the DP group included *Bifidobacterium longum*, *Bifidobacterium pseudocatenulatum*, *Blautia luti*, *Blautia glucerasea*, *Blautia provencensis*, *Corynebacterium dentalis*, *Enterocloster bolteae*, *Enterocloster clostridioformis*, *Erysipelatoclostridium ramosum*, *Faecalibacterium duncaniae*, *Faecalibacterium prausnitzii*, *Fusicatenibacter saccharivorans*, *Gemmiger gallinarum*, *Ruminococcus gnavus*, *Ruminococcus torques*, and *Streptococcus thermophilus* (Figure 5B). Conversely, the Healthy group was associated with *Agathobaculum butyriciproducens*, *Bacteroides kribbi*, *Blautia intestinalis*, *Clostridium saudiense*, *Dorea longicatena*, *Escherichia albertii*, *Faecalibacillus intestinalis*, *Faecalibacterium hattori*, *Gemmiger formicilis*, *Lactobacillus rogosae*, *Petroclostridium xylanilyticum*, *Phascolarctobacterium faecium*, and *Phocaeicola plebeius*. Bacteria were made 5 clusters in network analysis (Figure S6B). Interestingly, the abundance of gut bacteria showed negative associations between the Healthy and DP groups (Figure 5C). The bacteria were positively correlated within the same group.

Further network analysis using Minimal Common Oncology Data Elements (MCODE) with the same criteria as ET-B revealed that the total gut bacteria in ET-L clustered into five distinct clusters at the species level (Table 3). The darker color represents higher MCODE scores, indicating the primary bacterial cluster. Among the five clusters, the cluster with the highest MCODE score was the primary bacterial cluster, with *Petroclostridium xylanilyticum* as the seed bacteria belonging to the Healthy group. However, the rest of the bacteria were high in either the Healthy or DP groups. They showed positive and negative interactions with each other. The seed bacterium for the second cluster was *Bacteroides stercoris*, and the second cluster also included *Bacteroides eggerthii*, *Prevotella stercorea*, *Phocalcola plebius*, and others. They were also closely linked to the bacteria in the primary cluster. The interaction of the bacteria in ET-L indicated that the gut bacteria community could not be easily disrupted.

In ET-L, the average degree and path length indicating microbial interconnections were much higher in the DP group than in the Healthy group, and the stability of the network, represented by negative nodes, was not significantly different between the Healthy and the DP groups (Table 2). These results suggest that the gut bacteria in the Healthy and DP groups could not be altered.

### 2.7. Metagenome Function in ET-B

**Figure 6:**
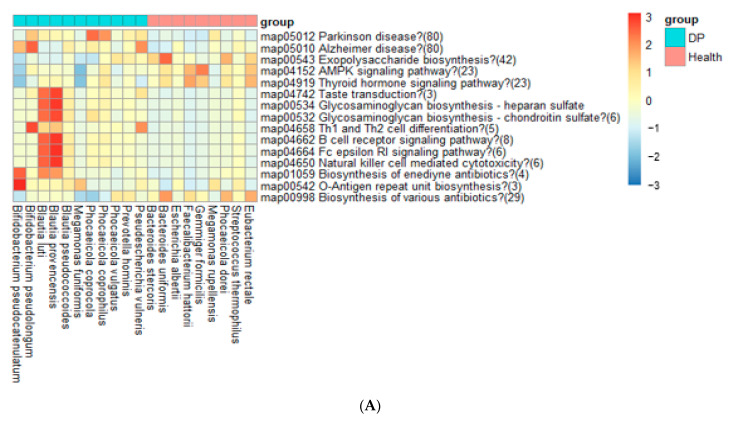

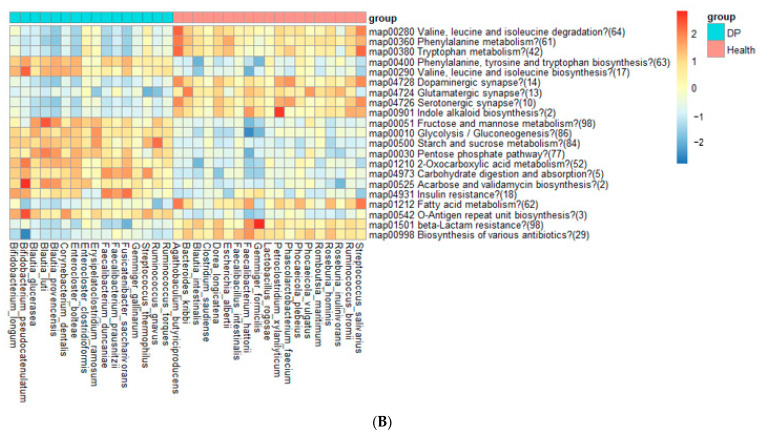
Metagenome functions of the primary gut microbiota according to each enterotype. (**A**) ET-B. (**B**) ET-L.

The metagenome function analysis of the gut bacteria revealed distinct profiles and contrasting associations between the DP and Healthy groups. The functions linked to Parkinson’s disease, Alzheimer’s disease, taste transduction, glycosaminoglycan biosynthesis, and immune-related processes were positively associated with the DP group but negatively associated with the Healthy group (Figure 6A). Conversely, the AMP-activated protein kinase (AMPK) signaling pathway, the thyroid hormone signaling pathway, and exopolysaccharide biosynthesis were positively associated with the Healthy group and negatively associated with the DP group. Notably, the differences in intensity of metagenome function between the Healthy and DP groups were less pronounced in ET-B than in ET-L.

### 2.8. Metagenome Function in ET-L

The comparison of metagenome functions between the Healthy and DP groups revealed significant differences. The metabolic pathways related to glucose, amino acids, fatty acid metabolism, and neurotransmitter metabolism were found to have statistically significant differences. Specifically, the following exhibited a positive correlation with the Healthy group and a negative correlation with the DP group: valine, leucine, and isoleucine degradation; phenylalanine and tryptophan metabolism; and indole alkaloid biosynthesis (Figure 6B). Conversely, the biosynthesis of valine, leucine, isoleucine, phenylalanine, and tryptophan showed a negative association with the Healthy group and a positive association with the DP group.

In neurotransmitter pathways, the dopaminergic, glutaminergic, and serotonergic synapses were positively linked to the Healthy group and negatively associated with the DP group. However, the pathways related to fructose and mannose metabolism, glycolysis/gluconeogenesis, carbohydrate digestion and absorption, the pentose phosphate pathway, and insulin resistance were negatively associated with the Healthy group and positively related to the DP group. Interestingly, immune-related pathways, such as O-antigen repeat unit biosynthesis, beta-lactam resistance, and antibiotic biosynthesis, showed opposing influences in the Healthy and DP groups.

## 3. Discussion

The gut–brain axis refers to the bidirectional communication between the central nervous system (brain and spinal cord) and the gut and involves multiple neural, hormonal, and immune signaling pathways [15]. Growing evidence suggests that disruptions in the gut–brain axis may contribute to the development and progression of several disease conditions, including depression [16]. However, it is unclear whether microbial dysbiosis is linked to the pathology of depression [17]. The present study aimed to investigate alterations in gut bacterial compositions and metagenome functions in patients with depression compared to those in healthy Chinese individuals according to each enterotype. In this study, the gut bacteria in ET-L and ET-B were separated and distinct in the Depressed and Healthy groups, but the α-diversity was not significantly different between the two groups. The bacteria in the ET-L group were a more tightly interconnected microbial community than those in the ET-B group. The metabolic pathways related to glucose metabolism, amino acid degradation, neurotransmitter metabolism, and immune-related functions showed strong positive associations with depression, particularly within ET-L. In contrast, the associations were less pronounced in ET-B. Furthermore, vagotomy suppressed depression symptoms, suggesting that the vagus nerve is involved in depression through the gut–brain axis [18]. These findings provide insights into the relationship of the gut–brain axis in the pathogenesis of depression in Asian individuals.

Scientific interest in the relationship between gut microbiota and depression is growing [15]. While the exact mechanisms are still being studied, several known aspects suggest a connection between gut microbiota and depression [17]. They include gut microbiota dysbiosis involving *Atopobium*, *Enterobacteriaceae*, and *Subdoligranulum*, which are associated with depression and are believed to be involved in neurotransmitter production based on data from earlier studies [19]. Among the gut bacteria, *Faecalibacterium* and Ruminococcaceae are inversely linked to depression symptoms via serotonin and GABA production, short-chain fatty acid production, and anti-inflammatory activity [20]. The other bacteria are positively correlated with inflammatory activity and promote leaky gut syndrome. The present study exhibited consistent results in all patients with depression, wherein they exhibited a decrease in *Faecalibacterium* and an abundance of *Eggertherlia*, *Erysipeliatoclostridium*, and *Enterocloster*. At the species level, *Lachnoclostridium pancanse*, *Facalibacllilus intestinalis*, *Rombousia martimum*, and *Faecalibacterium hattori* were higher in all participants in the Healthy group, and *Erysipelactoclostrodium ramosum, Enterocloster bolteae*, *Blautia luti*, *Blautia glucerasea* were higher in the DP group. However, the gut microbiota relevant to DP remains inconsistent [21].

Due to the complexity of the gut microbiota, it is better to classify them into enterotypes to study their relationship with disease [22]. Host genetics and lifestyle, mainly dietary patterns, determine a person’s enterotypes, and the incidence of certain diseases is closely related to enterotypes [22]. Gut microbiota has different networks to modulate their growth, and the alteration of each network influences depression incidence differently [23]. A few studies have explored the relationship between depression and gut microbiota according to enterotypes. In Korean adults, α-diversity measured with the Shannon index is positively associated with the positive emotion measured by the positive affect negative affect schedule (PANAS), a self-reported measure, in ET-P but not in ET-B [24]. In the present study, the participants’ gut microbiota could be separated into three enterotypes: ET-L, ET-B, and ET-P. The β-diversity of the gut microbiota was significantly separated between the DP and Healthy groups in ET-L but not in ET-B or ET-P. Since the ET-P group did not have sufficient participants, ET-B and ET-L were used to compare the gut microbiota between the DP and Healthy groups. In ET-L, *Blautia provencensis*, *Blautia luti*, *Blautia glucerasea, Collinsella aerofaciens*, *Enterocloster bolteae*, *Enterocloster clostridioformis*, and *Ruminococcus gnavus* were selected for the DP group using ALDEx2, and they were significantly higher in the DP group than in the Healthy group at *p* < 0.0001 (Bonferroni correction). However, no bacteria were selected for the DP group by ALDEx2 in ET-B. *Pserudescherichia vulneris*, *Petroclostridium xylanilyticum*, and *Clostridium innocuum* had a higher LDA score in the DP group of ET-B. The bacteria in the Healthy group were similar in ET-L and ET-B. Therefore, in ET-L adults, the depression status could be modified by altering the gut microbiota.

Previous studies have shown that individuals with irritable bowel disease (IBD) are more likely to experience symptoms of depression, anxiety, and other mood disorders than the general population [25]. The gut microbiota has emerged as a potential link between depression and IBD, and alterations in the composition and diversity of the gut microbiota are associated with depression and IBD [26]. The relationship between specific *Clostridium* and *Veillonella* species and depression is still an area of active research, and the exact mechanisms by which these bacteria may contribute to depression are not fully understood. *Clostridium* is a bacterial genus that encompasses several species, and certain species within this genus have been implicated in depression and IBD [27]. The present study shows that *Clostridium*, including *Clostridium porci* and *Clostridium innocuum*, was higher in the DP group than in the Healthy group in both ET-L and ET-B. Therefore, the abundance of some *Clostridium* species increases in patients with IBD and depression, suggesting that alterations in the gut–brain axis may be the underlying mechanism for both diseases.

Interestingly, *Bifidobacterium longum*, *Bifidobacterium adolescentis*, and *Faecalibacterium prausnitzii* are known as beneficial bacteria for anxiety and depression-related symptoms with reducing inflammatory cytokines [28,29]. However, in the present study, they belonged to the DP group and were tightly and negatively associated with the bacteria in the Healthy group, such as *Faecalibacterium hattori*, *Germmiger formicillis*, and *Blautia intestinalis*. In ET-B, some gut microbiota in the Healthy and DP groups were similar to those in ET-L, but some in ET-B were different from those in ET-L. In ET-B, unlike in ET-L, *Bacteroides uniformis*, *Phocaeicola dorei*, and *Bacteroides stercoris* were the primary bacteria in the Healthy group. The network characteristics primarily reflect the structural properties and connectivity patterns of the networks [30]. However, few studies have demonstrated the different gut microbiota networks in healthy individuals and patients with depression [14]. The gut bacterial interactions showed a more intricate microbial network in individuals with depression than in those in the Healthy group in ET-L rather than in ET-B in the present study. In ET-B, the microbial network and stability were higher in the Healthy group than in the DP group, but in ET-L, they were higher in the DP group than in the Healthy group. These results suggest that there could be a transition of the gut microbiota in the DP group to that of the Healthy group with prebiotics or probiotics treatment in ET-B. However, such a shift would be difficult in ET-L.

Gut permeability refers to the integrity of the intestinal lining, determined by zonulin-1 expression [31]. The intestinal lining serves as a barrier that selectively allows beneficial nutrients and molecules to pass through while preventing harmful substances, such as inflammatory cytokines and bacterial metabolites, from entering the bloodstream [32]. Emerging research suggests that disruptions in gut permeability may play a critical role in influencing mental health, including depression. An imbalanced gut microbiome involved in *Akkermansia muciniphila* can contribute to modulating gut permeability [32]. Changes in gut permeability and gut microbiome composition can influence vagal nerve signaling [33]. This signaling pathway plays a role in regulating mood, stress responses, and cognitive function [34]. Gut microbiota is involved in producing and regulating neurotransmitters, metabolites, and inflammatory cytokines, which alter gut permeability to modulate depression and cognitive function through gut–brain axis communication [31,32,33].

The metagenome function represents the collective genetic information and functional capabilities of the entire gut microbial community. Recent studies have explored the role of metagenome function related to depression: the gut microbial metagenome is linked to neurotransmitter synthesis and metabolism, immune system modulation, and inflammation through modulating the gut–brain axis [35]. The present study also showed that the metabolic pathways related to glucose metabolism, amino acid degradation, neurotransmitter metabolism, and immune-related functions had negative associations with depression, particularly within ET-L. Since amino acid degradation, including that of tryptophan and tyrosine, is associated with neurotransmitter production, the results of metagenome function related to amino acid metabolism suggested decreased neurotransmitter production in the DP group, as shown in previous studies [36]. Interestingly, carbohydrate digestion and absorption, fructose, mannose and glucose metabolism, and the pentose phosphate pathways were positively associated with the DP group, indicating that the gut bacteria in the DP group utilized glucose well, which, in turn, increased insulin resistance in the host. This could be linked to carbohydrate cravings in people with depression. Carbohydrate craving is reported to be connected to low serotonin levels in people with depression [37]. Therefore, the symptoms of depression may be a result of the gut microbiota composition and its interaction with the gut–brain axis.

The impact of diet on alleviating depression and its influence on gut microbiota composition represent critical aspects for comprehending the intricate interplay between dietary choices and the equilibrium of gut microbiota [38]. While addressing specific individual bacteria associated with dietary patterns to mitigate depression risk presents challenges within the current scientific understanding, the present study establishes an association between gut microbiota and impaired glucose metabolism characterized by increased insulin resistance. It underscores the significance of adopting diets that ameliorate insulin resistance to enhance depression risk reduction potentially [39]. Particularly notable is the recommendation for individuals, especially those with ET-L, to embrace diets low in simple sugars and saturated fats while incorporating foods rich in soluble dietary fiber. The Mediterranean diet, celebrated for its emphasis on whole foods, legumes, nuts, healthy fats, and various fruits and vegetables, has garnered attention for its potential to nurture a diverse and advantageous gut microbiota profile [38]. This dietary approach could potentially contribute to improved depression risk outcomes. However, further investigation is warranted to pinpoint specific dietary fiber types or probiotics that could effectively diminish the risk of depression, tailored to the distinctive profiles of specific enterotypes.

The present study was novel in several aspects: (1) The network analysis of a more tightly interconnected microbial community within ET-L than within ET-B suggests potential functional relationships among gut microbiota that are stronger within ET-L. (2) The gut microbiota were well separated in ET-L compared to in ET-B. (3) The metabolic pathways related to glucose metabolism, amino acid degradation, neurotransmitter metabolism, and immune-related functions showed strong negative associations with depression, particularly within the ET-L enterotype. These findings contribute to our understanding of the gut–brain axis and its role in the pathogenesis of depression, thus highlighting the potential for precision medicine interventions. The limitations of the study are as follows: (1) The data were collected from cross-sectional studies. (2) Although all available amplicon data of gut microbiota were collected, the sample size was still insufficient (333 participants, with 226 individuals in the DP group). The gut characteristics of depression in ET-P could not be studied due to the small sample size. (3) Potential confounding factors influencing the gut microbiota and depression, such as diet, comorbidities, or lifestyle factors, were not provided and could not be adjusted for the analysis. However, since we did not include the FASTA/Q data of depression medication users, the depression medication, a major confounding factor, was eliminated. Additionally, the effects of dietary patterns and host genetics were partly partially attenuated when segregating the participants according to enterotype in the present study.

In conclusion, this study revealed the connection between gut microbiota and depression in Chinese adults. The research shows distinct differences in microbial composition within the ET-B and ET-L in Chinese individuals with depression. Specific bacterial genera and species were found to be more prevalent in the depressed groups of both enterotypes (ET-B and ET-L). Noteworthy bacteria like *Mediterraneibacter*, *Blautia*, *Bifidobacterium*, and *Clostridium* were elevated in the DP group of ET-B, while Bifidobacterium, *Blautia*, *Clostridium*, *Collinsella*, and *Corynebacterium* were prominent in the DP group of ET-L. The analysis also identified key metabolic pathways linked to depression, including glucose and neurotransmitter metabolism, showing negative associations, particularly in ET-L, suggesting a potentially stronger functional relationship among gut bacteria in this enterotype. In the metagenome function analysis, the metabolic pathways related to glucose metabolism, amino acid degradation, neurotransmitter metabolism, and immune-related functions exhibited negative associations with depression, particularly within ET-L. These findings provide insights into the role of the gut–brain axis in depression, particularly in Asian individuals. The observed microbiota shifts and functional differences hold potential for diagnostic and therapeutic advancements. However, further research is required to fully comprehend the mechanisms behind gut microbiota and depression, offering new avenues for precision medicine interventions.

## 4. Methods and Materials

### 4.1. Collection of FASTA/Q Files of Fecal Bacteria from Depressed and Healthy Adults

The collection of FASTA/Q files of fecal bacteria from adults with and without depression was conducted using a specific selection process, which is outlined in Figure 1. The files were obtained from various databases, including the National Center for Biotechnology Information (NCBI, Bethesda, MA, USA), European Nucleotide Archive (ENA), and the data repository for Gut Microbiota (GMrepo, Cambridge, UK), and they included data available until April 2023. The FASTA/Q files were selected from studies based on the following inclusion criteria: human host (Homo sapiens), target participants (depressed and healthy Chinese adults over 30 years old), sample type (human feces), assay (amplicon sequencing—Miseq), and target sequencing (16S rRNA) (Figure 1). Depression often occurs during the adolescent years or early adulthood despite developing at any age [40]. However, the classic symptoms of depression, such as persistent sadness, a loss of interest, changes in appetite or sleep patterns, fatigue, and feelings of worthlessness, are often prominent in adults [40]. The study target was adults. All the included studies had obtained approval from institutional review boards and informed consent from participants who volunteered to provide fecal samples. Each study obtained informed consent from all subjects.

A total of 333 fecal FASTA/Q files were collected from the studies (Table 1), but they did not provide comprehensive data on demographics and lifestyles. Age and gender information was available for only a subset of participants, with an average age of approximately 43 years and an almost equal distribution of genders.

### 4.2. Gut Microbiota Composition and Community Analysis

The downloaded FASTA/Q files of fecal bacteria from humans were processed using the NCBI Sequence Read Archive toolkits (https://trace.ncbi.nlm.nih.gov/Traces/sra/sra.cgi?view=software (accessed on 2 November 2022)). DNA sequences from the fecal samples were extracted and obtained as FASTA/Q files. The sequences underwent processing and clustering using a 97% similarity threshold to collect the operational taxonomic units (OTUs) as described in previous studies [10]. OTUs were annotated using the NCBI Basic Local Alignment Search Tool (BLAST) for taxonomy assignment (https://blast.ncbi.nlm.nih.gov/Blast.cgi (accessed on 6 December 2022)). A total of 2647 representative sequences were obtained, and their biome files, containing taxonomy and counts, were utilized for further analysis.

### 4.3. Enterotype Classification

Enterotypes were identified through PCA using gut microbiota from the collected fecal FASTA/Q files. The determination of the number of enterotypes was based on eigenvalues >1.5, using the “FactoMineR” and “Factoextra” packages in R software 4.2.2 [13]. The optimal number of clusters was found to be 3, resulting in the assignment of three enterotypes. These enterotypes were named based on the predominant bacteria at the family level: ET-B, ET-L, and ET-P. Distinct gut microbiota associated with depression risk were identified according to ET-B, ET-L, and ET-P.

### 4.4. Diversity and LDA Scores of Gut Microbiota

In each enterotype, the participants were categorized into Healthy and DP groups, and the DP-linked bacteria and metagenome function were determined. The composition and diversity of gut bacteria are vital for host metabolism and overall health status, including depression. α-diversity describes the mean species diversity within an individual’s gut and was quantified using the Chao1, Shannon, and Simpson indices. These metrics were calculated using the “summary.single” command in the mothur software version 1.48.0 package. β-diversity, which captures the differentiation between groups based on regional and local species diversity, was evaluated using the clearcut command in mothur to construct a phylogenetic tree. The unweighted UniFrac distance matrix was computed using the “unifrac.unweighted” command, followed by a principal coordinate analysis (PCoA) for visualization. The PCoA analysis effectively clustered the FASTA/Q samples into distinct Healthy and DP groups. The statistical differences between these groups were assessed using a permutational multivariate analysis of variance (PERMANOVA). Furthermore, the effect sizes of individual abundant species were determined using the LDA scores and analyzed using the LEfSe command in the mothur program. The gut bacteria representing the Healthy and DP groups were also determined with ALDEx2 in the R package.

### 4.5. XGBoost Classifier Training and SHapley Additive exPlanations (SHAP) Interpreter

A machine learning approach was employed with XGBoost, random forest, and linear regression to investigate the specific predominant gut microbiota associated with depression according to enterotype. The fecal data were randomly split into 80% for training and 20% for testing. The best hyperparameter settings were determined through a random grid search with 1000 iterations of the XGBoost algorithm using the Scikit package [10], generating the best model for distinguishing the Healthy and DP groups. The performance of the model was evaluated using the area under the AUROC on both the training and test sets. The 10-fold cross-validation was calculated using the cross_val_score function on the test data set, demonstrating an accuracy of 90% [11].

A SHAP analysis was performed on the output of the XGBoost model to identify the bacteria positively associated with the Healthy and DP groups [10,11]. The SHAP (0.39.0) package was utilized to provide the SHAP value of each bacterium to the classifier.

### 4.6. Network and Metagenome Function of Gut Microbiota

A correlation analysis at the species level was conducted using the sparse correlations for compositional data (SparCC) command in mothur. Microbiota with no significant differences and correlations below 0.1 were excluded. The species co-occurrence network (SCN) was visualized using the Cytoscape 3.4.0 application (https://cytoscape.org/ (accessed on 16 March 2023)). Gut microbiota were clustered with MCODE in Cytoscape. The complexity and stability of the gut microbiota network were calculated using the R package “igraph”.

The association between gut commensal bacteria and metabolic functions was predicted using the phylogenetic investigation of communities by reconstruction of unobserved states (PICRUSt2 version 2.0) software, and a correlation heatmap was generated using the pretty heatmap (Pheatmap, 1.0.12 version) R package. The metabolic functions of the genes within the gut microbiota were estimated using the Kyoto Encyclopedia of Genes and Genomes (KEGG) Orthologues (KO) and mapped using the KEGG mapper tool (https://www.genome.jp/kegg/mapper/search.html (accessed on 19 March 2023)) [23].

### 4.7. Statistical Analysis

A statistical analysis was performed using SAS version 7 (SAS Institute; Cary, NC, USA) and the R package. The data are presented as mean ± standard deviation (SD), and statistical significance was defined as *p* < 0.05. The mean differences between the Healthy and DP groups were assessed using a two-sample *t*-test. Data visualization was performed using R-studio and the ggplot2 package.

## Figures and Tables

**Table 1:** Fecal bacteria in clusters made with MCODE in the ET-L.

	Mean	Group	MCODE	Degree Layout	*p* Value	
Cluster 1						
*Corynebacterium dentalis*	0.457	DP	25.22	46	8.36 × 10^−5^	
*Collinsella aerofaciens*	1.302	DP	27.69	73	3.57 × 10^−7^	
*Blautia provencensis*	2.847	DP	25.28	58	1.41 × 10^−11^	
*Gemmiger gallinarum*	0.952	DP	26.18	45	1.77 × 10^−5^	
*Faecalibacterium prausnitzii*	0.636	DP	30.12	70	1.82 × 10^−5^	
*Faecalibacterium duncaniae*	1.403	DP	27.51	60	1.39 × 10^−7^	
*Anaerostipes hadrus*	0.981	Health	29.17	48	2.68 × 10^−8^	
*Blautia glucerasea*	1.283	DP	27.81	59	1.78 × 10^−10^	
*Ruminococcus gnavus*	1.867	DP	25.56	54	7.43 × 10^−7^	
*Petroclostridium xylanilyticum*	0.112	Health	30.94	60	0.00112	Seed
Cluster 2						
*Evtepia gabavorous*	0.184	DP	22.72	41	4.29 × 10^−5^	
*Blautia luti*	2.829	DP	23.44	54	2.21 × 10^−10^	
*Solibaculum mannosilyticum*	0.225	DP	24.62	51	9 × 10^−5^	
*Bacteroides stercoris*	0.963	DP	24.42	53	0.503	Seed
Cluster 3						
*Enterocloster bolteae*	0.396	DP	11.71	33	3.02 × 10^−9^	
*Bifidobacterium catenulatum*	1.072	Health	13.38	28	0.475	Seed
Cluster 4						
*Fusicatenibacter saccharivorans*	1.54	DP	10.45	33	3.01 × 10^−7^	
*Bifidobacterium pseudocatenulatum*	3.8	DP	10	19	2.35 × 10^−5^	
*Mediterraneibacter glycyrrhizinilyticus*	0.209	DP	10.5	32	0.058	Seed

**Table 2:** Complexity and stability of fecal bacteria according to the ET-L and ET-B.

	ET-L		ET-B	
	Health	Depression	Health	Depression
No. of node	196	285	166	163
No. of edge	3599	8454	2000	1273
Average degree	36.7	55.1	24.1	15.6
Average path length	0.777	1.13	1.08	1.16
Graphic density	0.188	0.209	0.146	0.0964
Clustering coefficient	0.306	0.355	0.252	0.183
Negative edge ratio	0.452	0.463	0.456	0.352

**Table 3:** Fecal bacteria in clusters made with MCODE in the ET-B.

	Mean	Group	MCODE	Degree Layout	*p* Value	
Cluster 1						
*Butyricicoccus_faecihominis*	0.14407	DP	11.93939		0.004319	
*Eggerthella_lenta*	0.380625	DP	12		0.007643	
*Clostridium_colinum*	0.538333	Health	12.90196		0.017457	
*Alistipes_putredinis*	0.800764	Health	16		0.39137	seed
Cluster 2						
*Bacteroides_stercoris*	0.962871	DP	10.59692		0.030355	seed
Cluster 3						
*Bacteroides_fragilis*	0.466068	DP	6.019048		0.012808	
*Haemophilus_parainfluenzae*	0.290275	Health	5.666667		0.230277	Seed
Cluster 4						
*Escherichia_albertii*	1.071582	Health	3.333333		0.028736	
*Akkermansia_muciniphila*	0.264366	DP	3.928571		0.889247	seed
Cluster 5						
*Ligilactobacillus_salivarius*	0.393043	Health	8.423529		0.087931	seed
*Collinsella_aerofaciens*	1.302479	DP	7.73975		0.078893	

## Data Availability

The authors confirm that the data supporting the findings of this study are available within the article and its Supplementary Materials. The FASTA/Q files for human studies were downloaded from the NCBI, and the data for the animal studies are available upon request to the corresponding author.

## Supplementary Materials

The following supporting information can be downloaded at https://www.mdpi.com/article/10.3390/ijms241713329/s1.
