# Supplementary material for: Gut Microbiota Alterations and Their Functional Differences in Depression According to Enterotypes in Asian Individuals
Source: Int J Mol Sci. 2023 Aug 28;24(17):13329. doi: 10.3390/ijms241713329 (PMC10487633; doi:10.3390/ijms241713329)

Supplementary Table

Table S1. Information on the studies included in the meta-analysis

Accession number	Design	Sample grouping	Country	Sampling	DNA extraction	16S region	Seq Tech
PRJNA838414	Case–control	PSD;26 Health: 30	China	NA	Fecal genomic DNA by the CTAB/SDS method.	V3-V4	Illumina MiSeq
PRJNA776170	Case–control	Health:44 MDD;24	China	Immediately frozen stool sample (2 g) at −80°C.	Fecal DNA by the QIAamp® DNA Stool Mini Kit	V3-V4	Illumina MiSeq
PRJEB23500	Case–control	Health:47 BD;72	China	NA	MiSeq 600 cycle v3 kit	V3-4	Illumina MiSeq
PRJDB10562	Case-control	Depression:246 with 3 point: antidepressant drug (no medication treatment)	China	NA	NA	NA	NA

PSD: Post-stroke depression; MDD: major depressive disorder; BD: depression with bipolar disorder; NA, non-applicable.

Table S2. Accuracy, sensitivity, specificity and precision in the prediction model generated with machine learning approach according to enterotypes

ET-B	AUROC	Accuracy	Sensitivity	Specificity	Precision	F1
XGBoost	0.810±0.003	0.807±0.002	0.935±0.002	0.632±0.005	0.776±0.003	0.844±0.002
Random forest	0.871±0.003	0.769±0.003	0.963±0.004	0.551±0.005	0.714±0.003	0.829±0.002
Liner regress	0.647±0.004	0.654±0.003	0.866±0.003	0.364±0.004	0.651±0.003	0.738±0.003
**ET-L**	**AUROC**	**Accuracy**	**Sensitivity**	**Specificity**	**Precision**	**F1**
XGBoost	0.934±0.001	0.828±0.002	0.961±0.002	0.499±0.005	0.825±0.002	0.886±0.001
Random forest	0.857±0.002	0.740±0.002	0.920±0.002	0.298±0.005	0.763±0.002	0.832±0.002
Liner regress	0.857±0.002	0.740±0.002	0.920±0.002	0.298±0.005	0.763±0.002	0.832±0.002

AUROC, area under the receiver operating characteristic.

Supplementary figures

Figure S1. Relative abundance of gut bacteria in family level according to each enterotypes

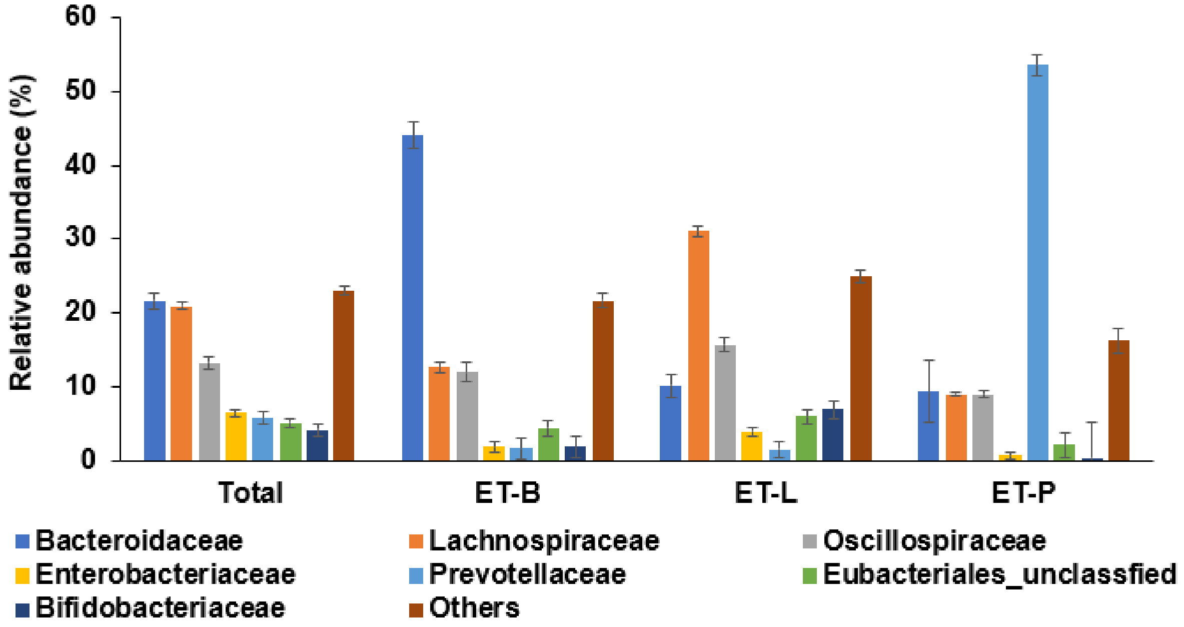

Figure S2. A-diversity of gut bacteria between the Healthy and Depressed (DP) groups according to enterotypes

A. All participants

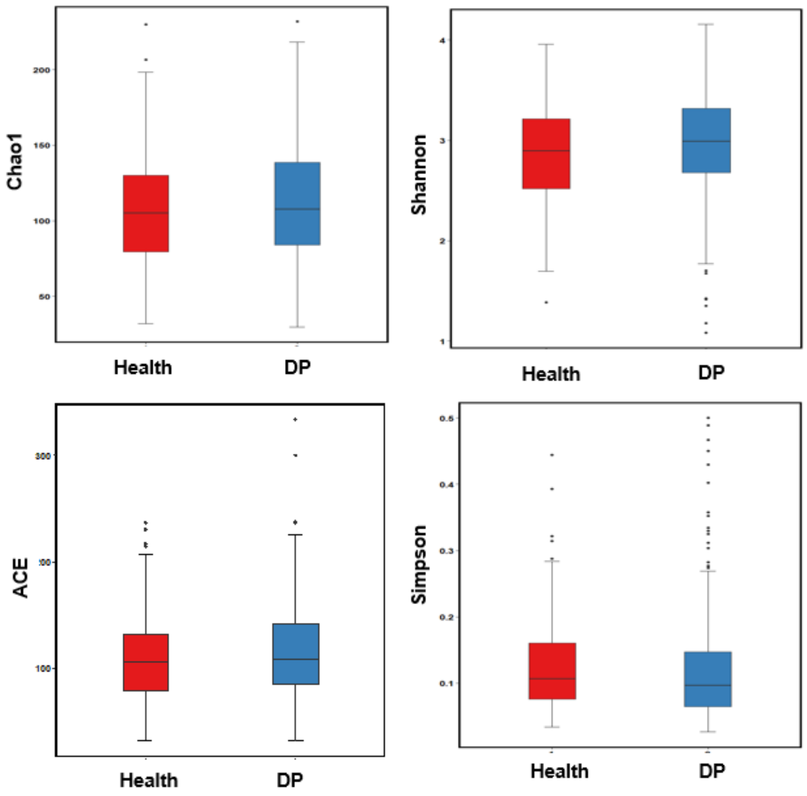

B. ET-B

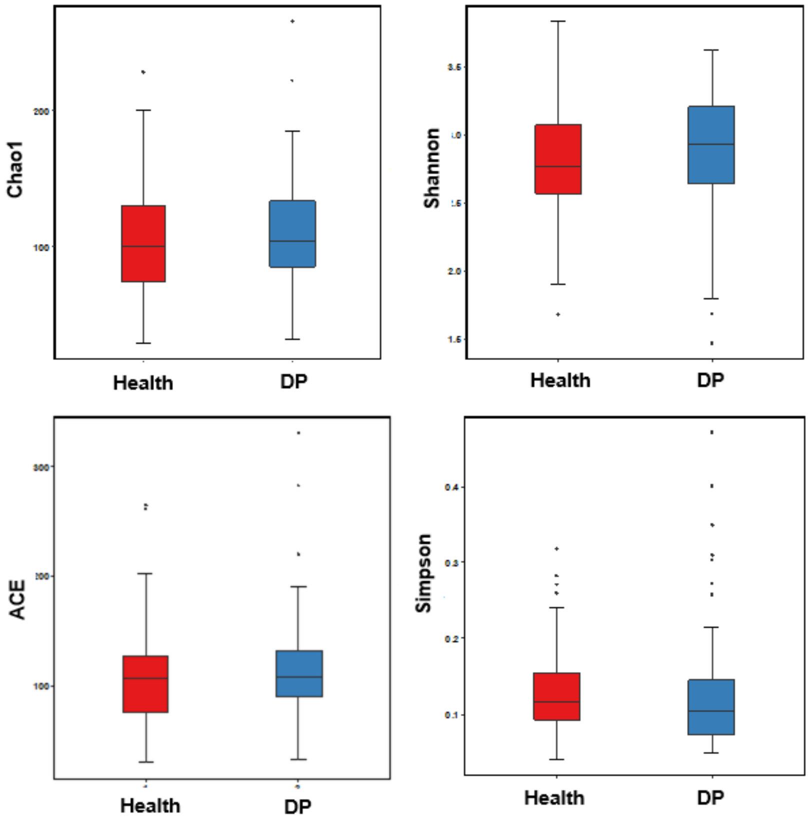
Chao1
Health
DP
Shannon
Health
DP
ACE
Health
DP
Simpson
Health
DP

C. ET-L

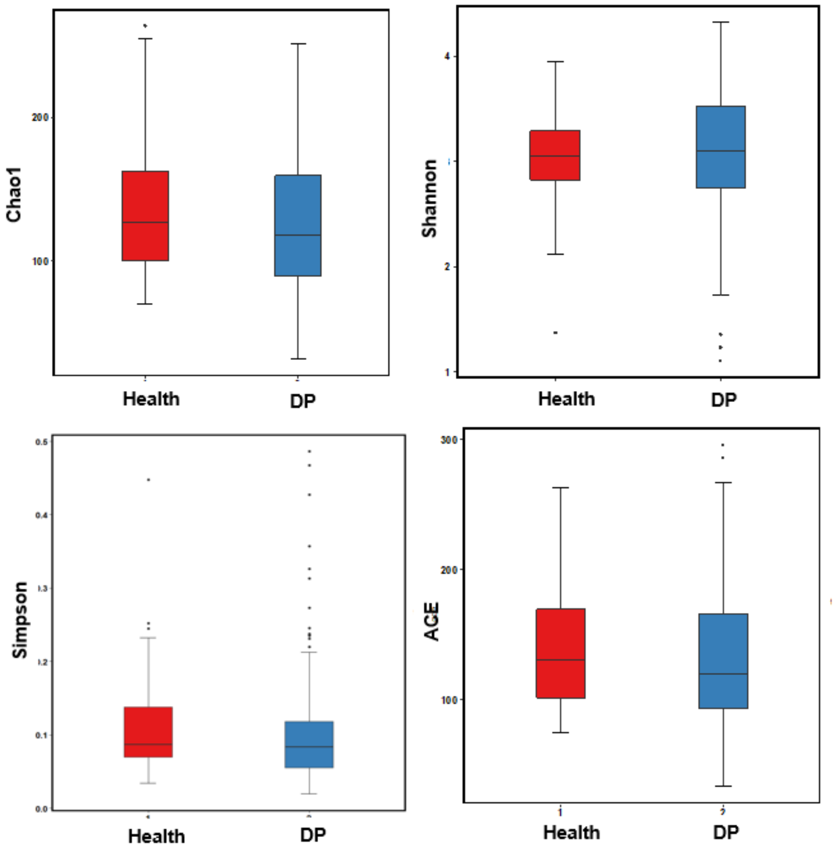
Chao1
Health
DP
Shannon
Health
DP
Simpson
Health
DP
ACE
Health
DP

D. ET-P

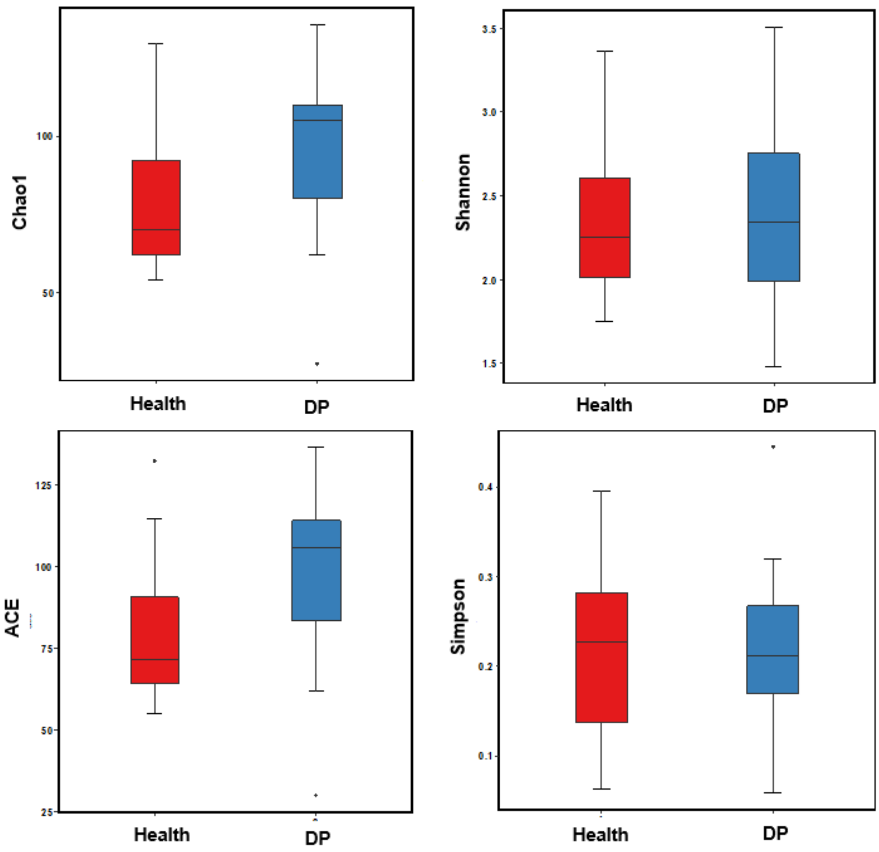
Chao1
100
50
Health
DP
Shannon
3.5
3.0
2.5
2.0
1.5
Health
DP
ACE
125
100
75
50
25
Health
DP
Simpson
0.4
0.3
0.2
0.1
Health
DP

Figure S3. B-diversity of gut bacteria between the Healthy and Depressed (DP) groups according to enterotypes

A. All participants

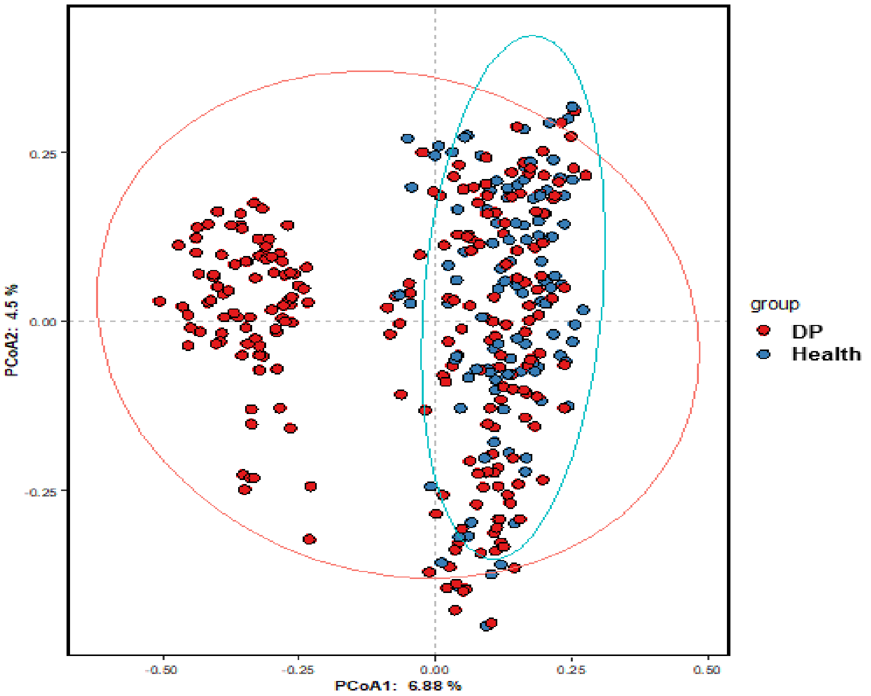

B. ET-B

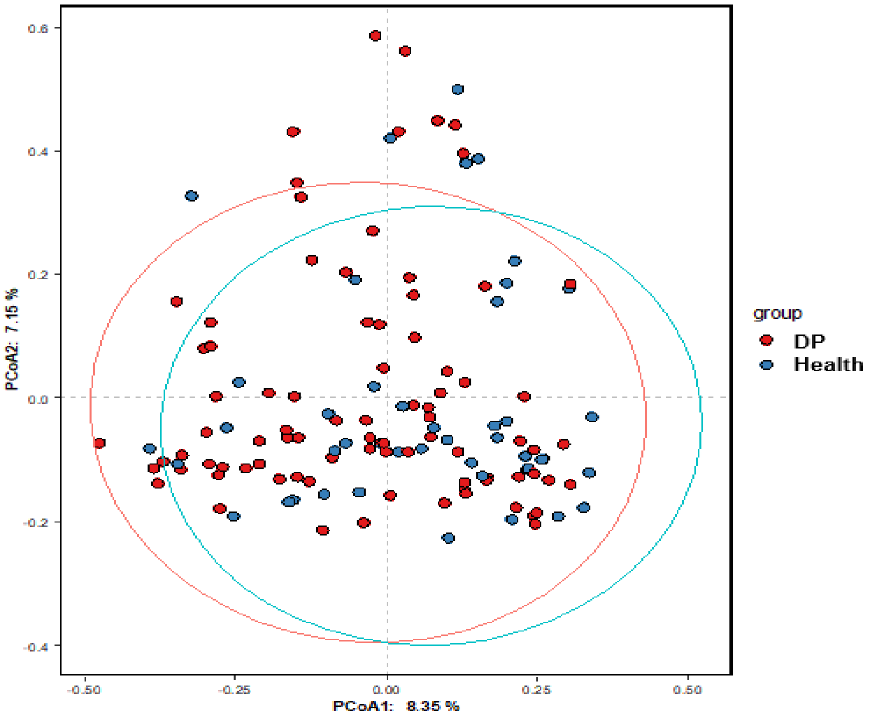

C. ET-L

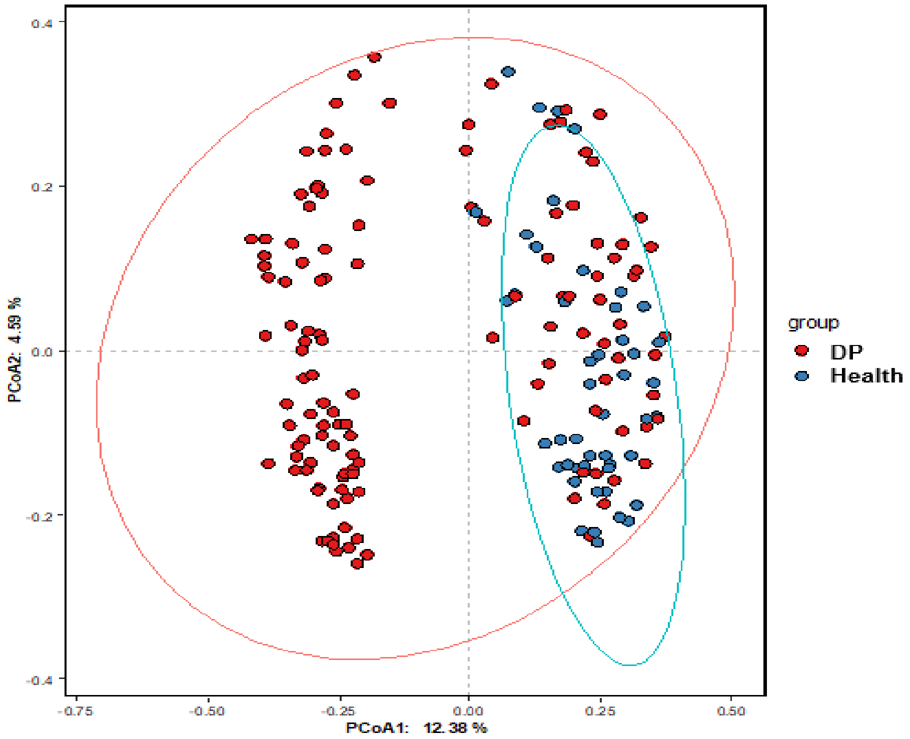
0.4
0.2
0.0
-0.2
-0.4
PCoA2: 4.59 %
-0.75
-0.50
-0.25
0.00
0.25
0.50
PCoA1: 12.38 %
group
DP
Health

D. ET-P

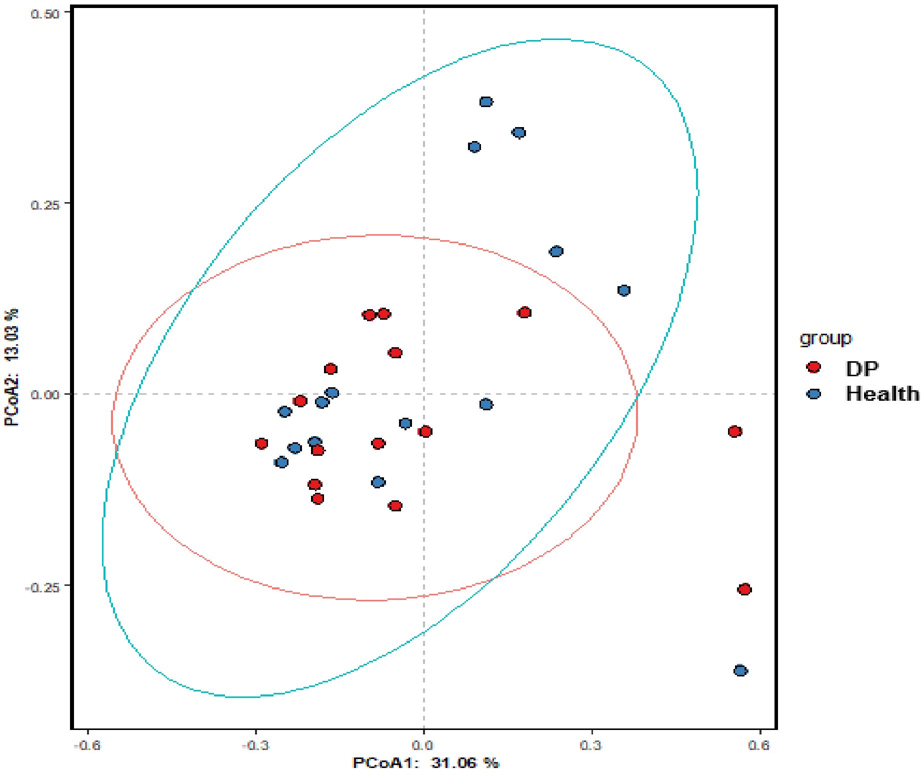
0.50
0.25
0.00
-0.25
PCoA2: 13.03 %
-0.6
-0.3
0.0
0.3
0.6
PCoA1: 31.06 %
group
DP
Health

Figure S4. Comparison of the gut microbiota compositions between the Healthy and Depressed (DP) groups in ET-P

A. Relative abundance of gut microbiota at the family level

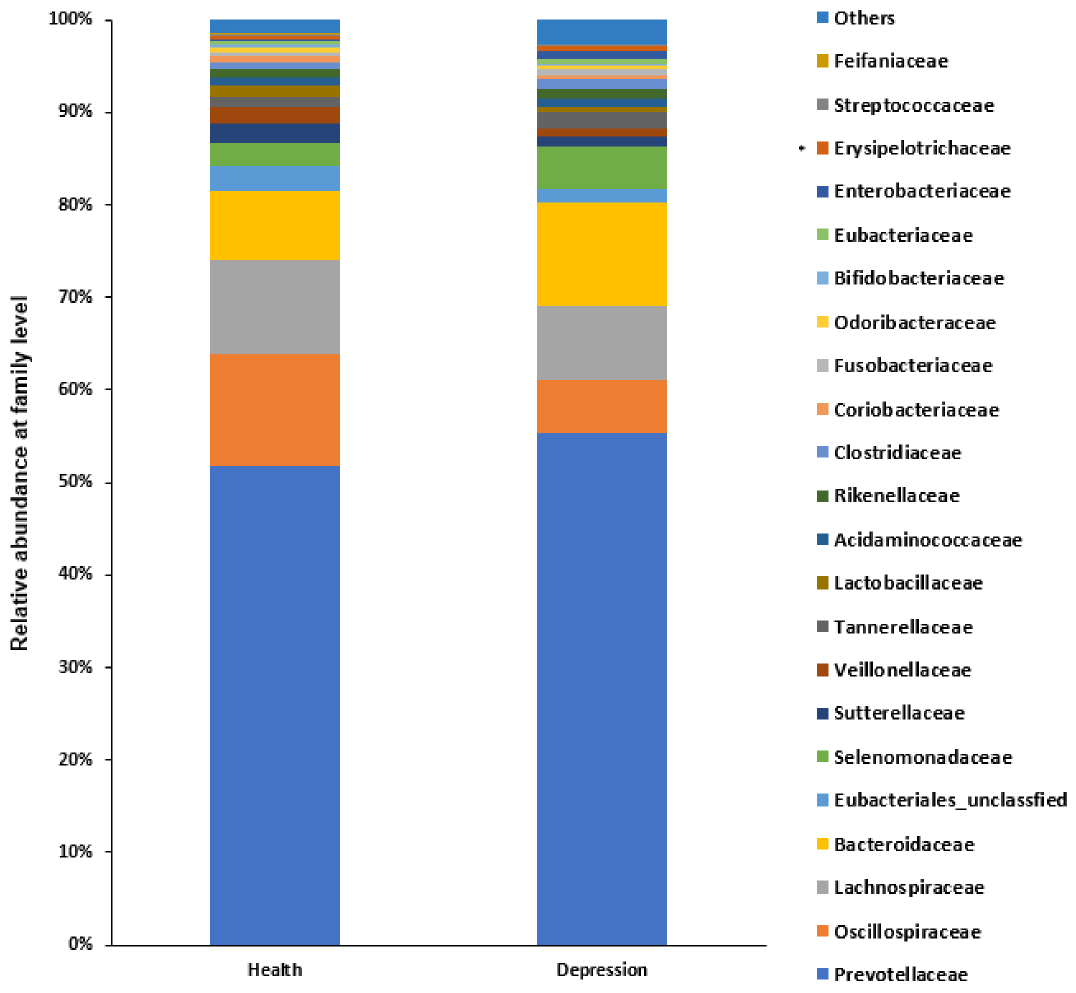

B. Relative abundance of gut microbiota at the genus level

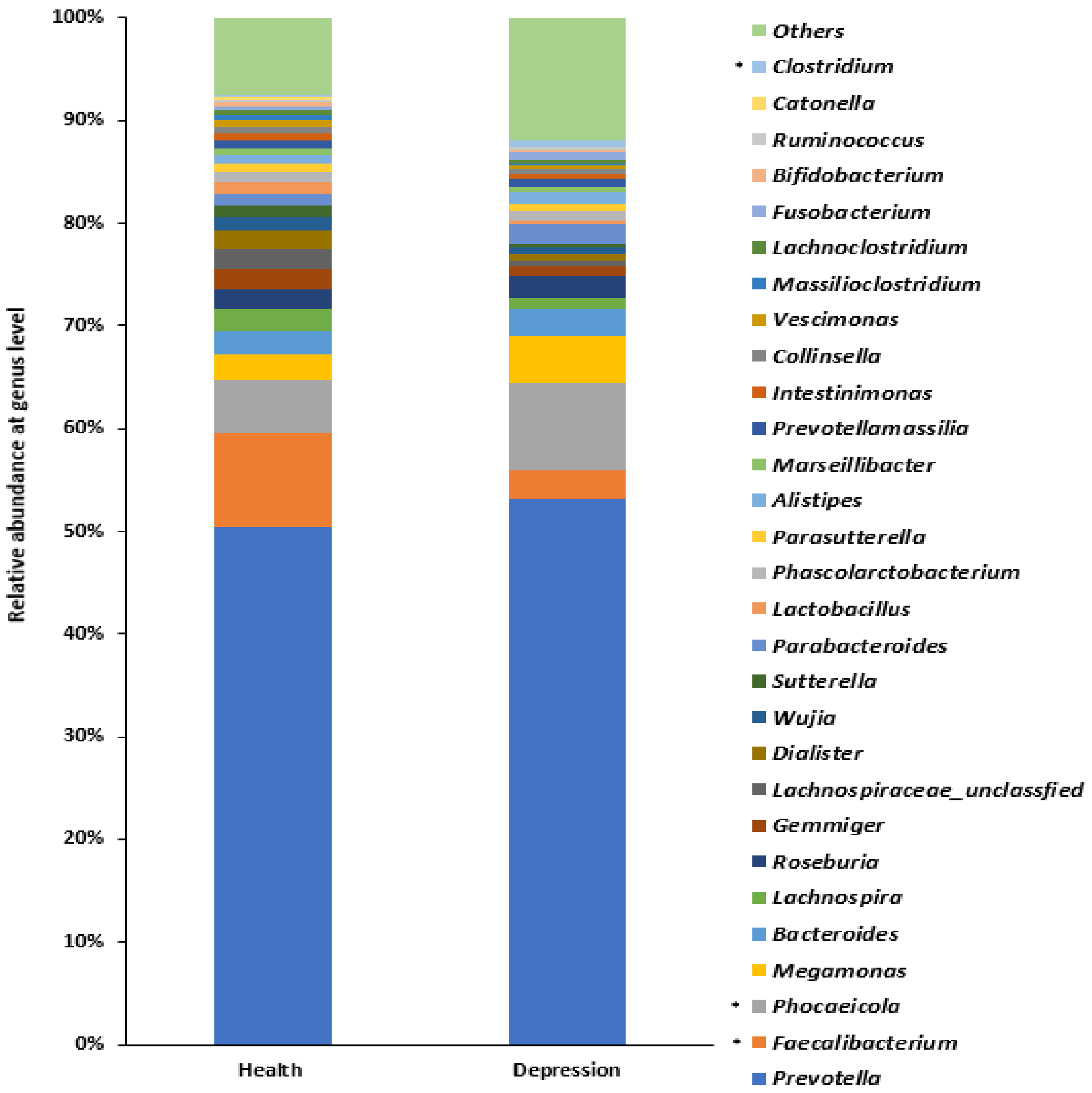

* Significant differences between the DP and Healthy groups at P<0.00001 (Bonferroni corrected P value).

Figure S5. Comparison of the gut microbiota composition between the Healthy and Depressed (DP) groups in all participants

A. Relative abundance of gut microbiota at the family level

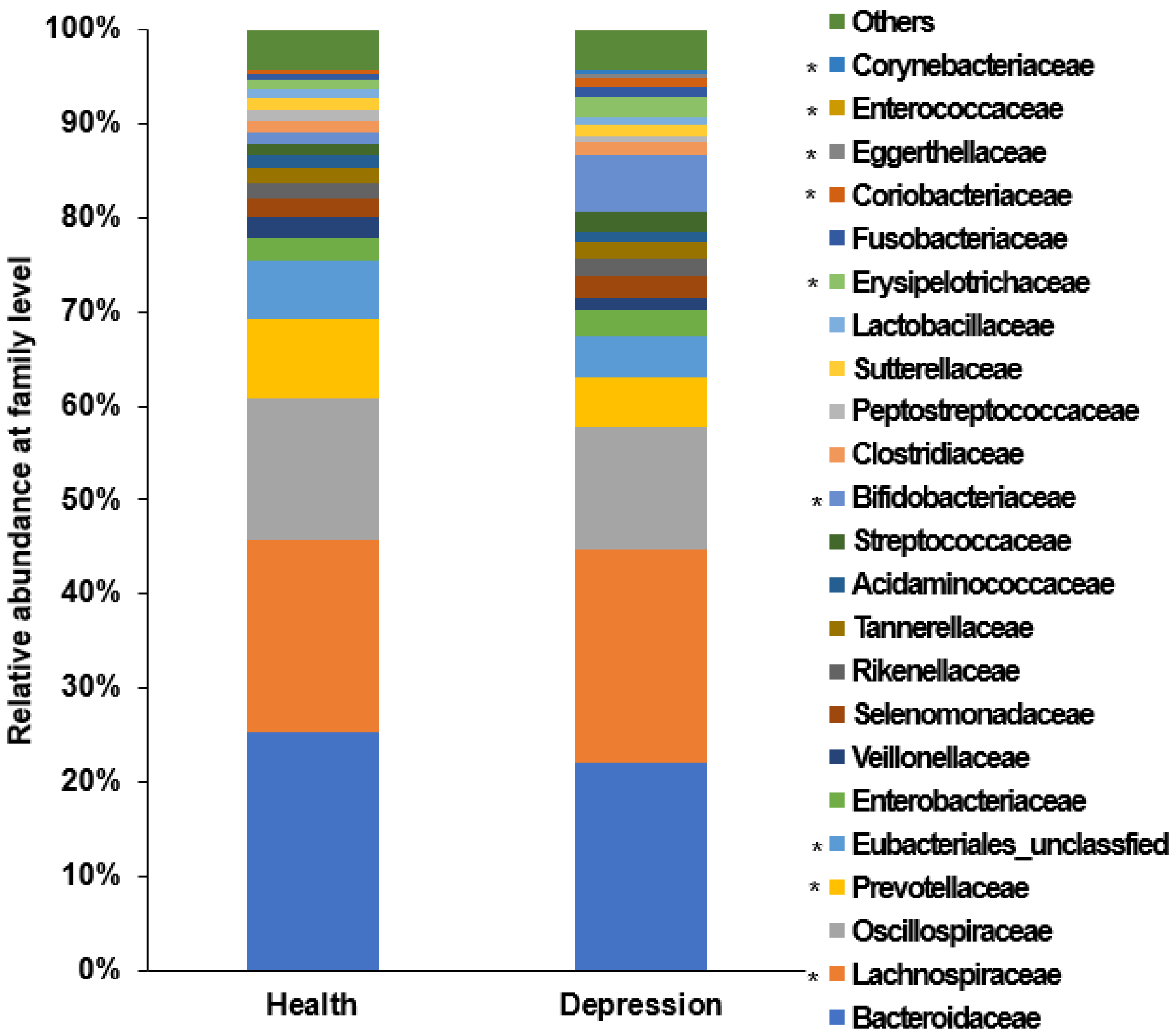

B. Relative abundance of gut microbiota at the genus level

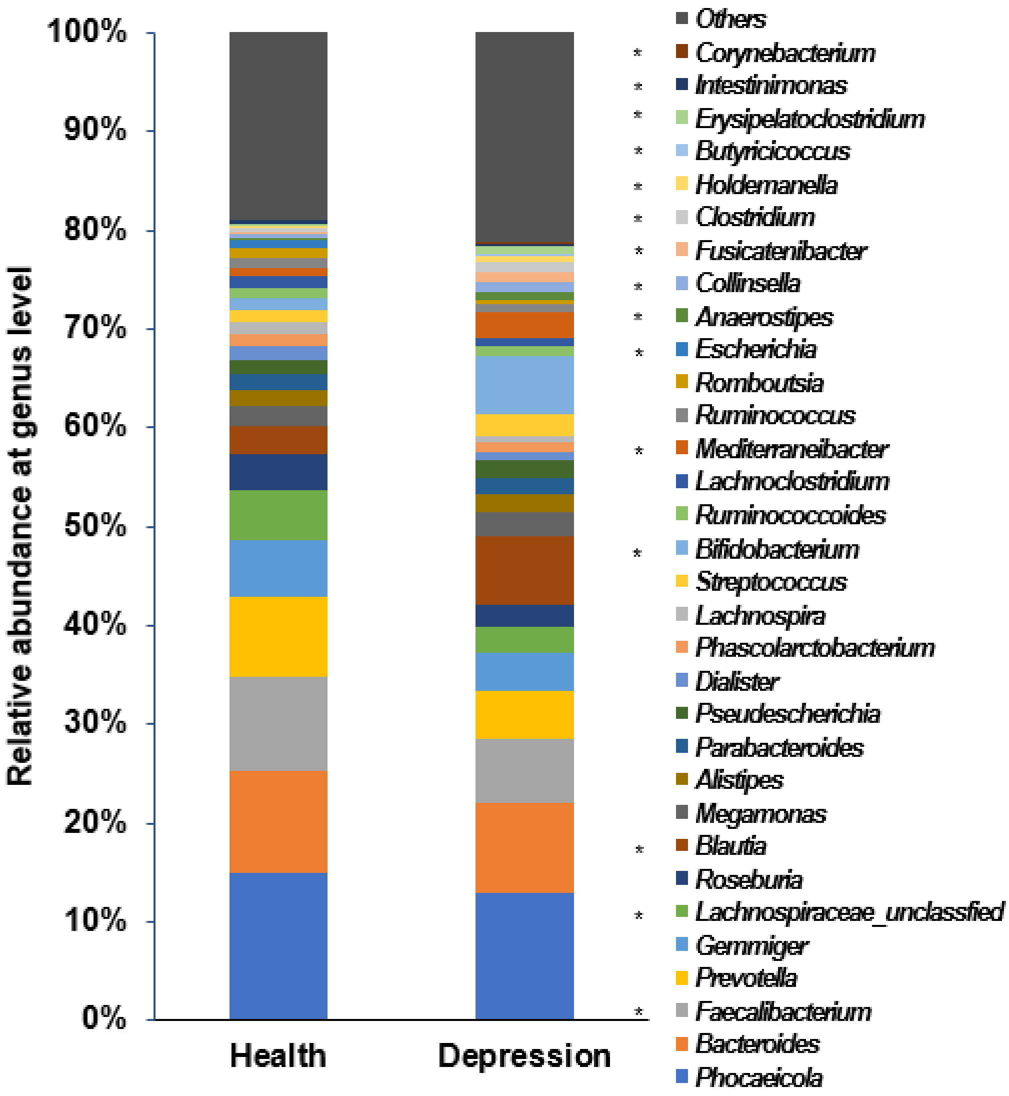

C. Primary gut microbiota in Healthy and DP groups at the species level in ET-B by ALDEx2

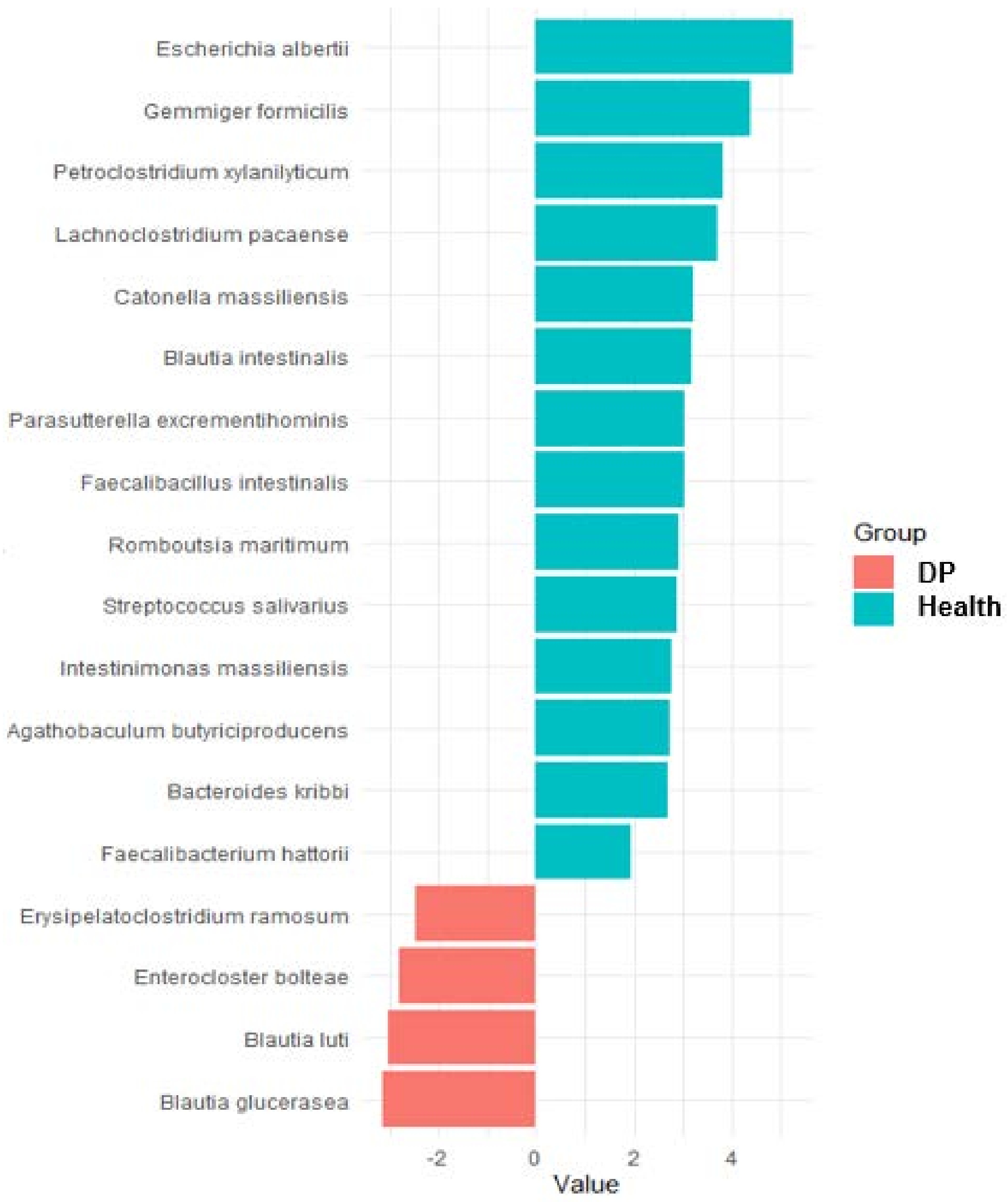

D. Primary gut microbiota in Healthy and DP groups at the species level in ET-B by linear discriminant analysis (LDA) scores

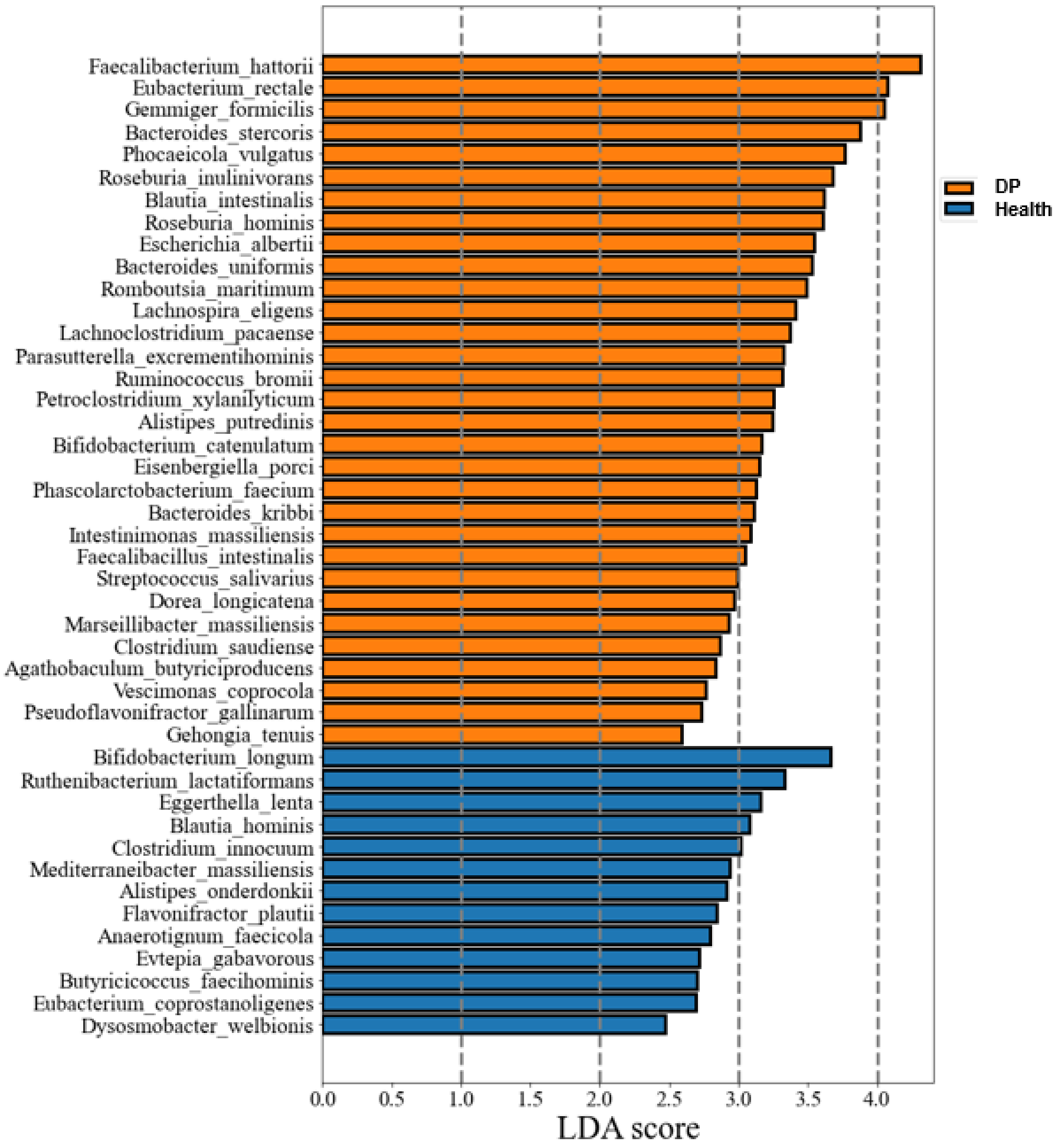

E. Area under receiver operating characteristic

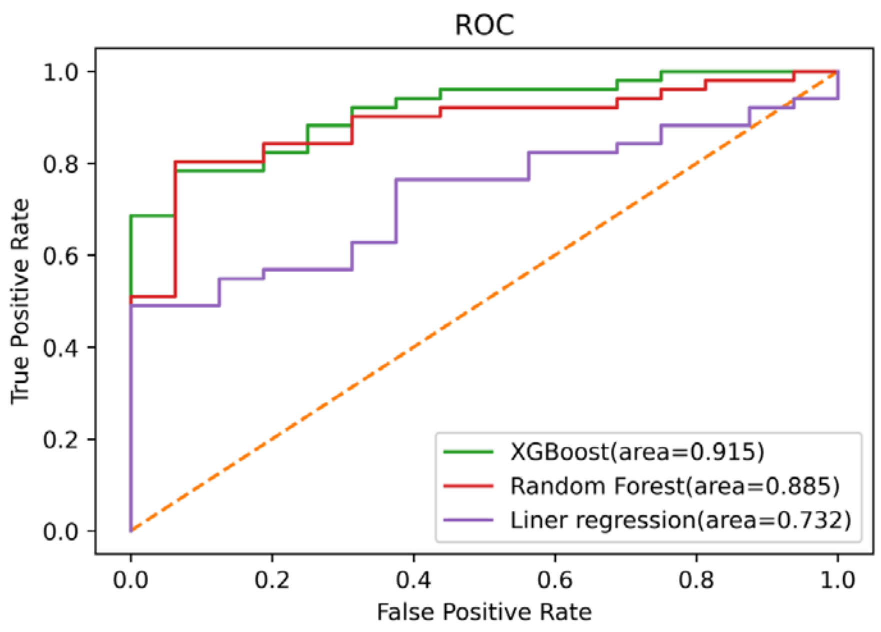

F. Primary gut microbiota in Healthy and DP groups at the species level by the XGBoost algorithm

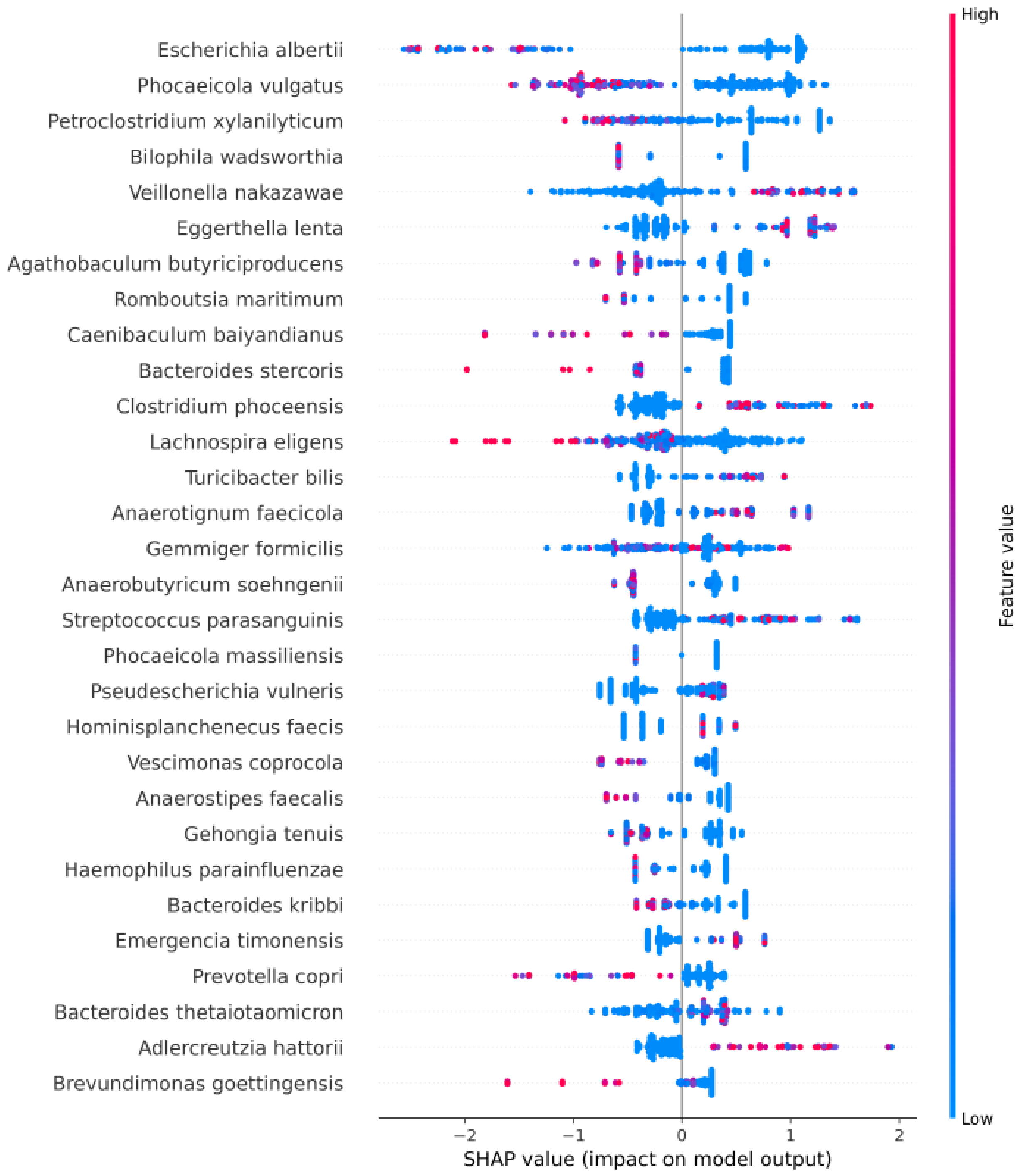

* Significant differences between the DP and Healthy groups at P<0.00001 (Bonferroni corrected P value).

Figure S6. Network of primary gut microbiota in the Healthy and Depressed (DP) groups in ET-B and ET-L

A. Clusters in ET-B

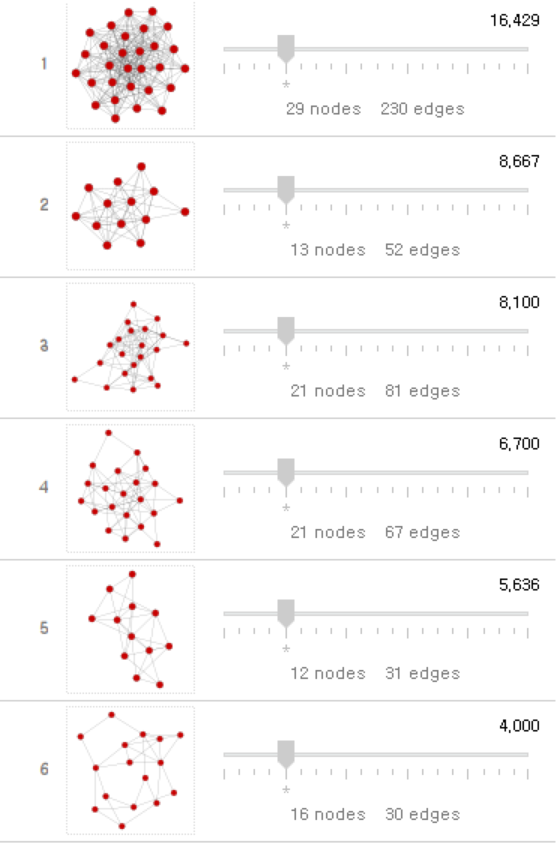

B. Clusters in ET-L

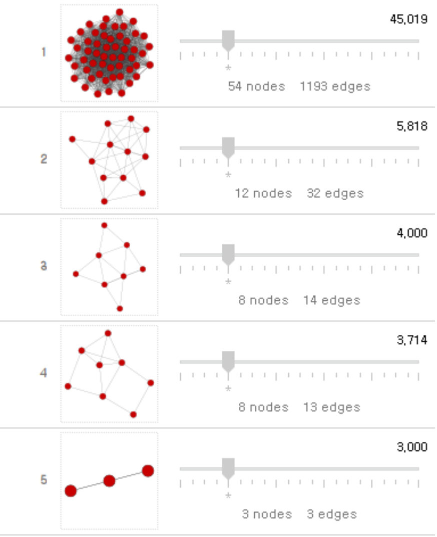

Figure S6. Network of primary gut microbiota in the Healthy and Depressed (DP) groups in all participants

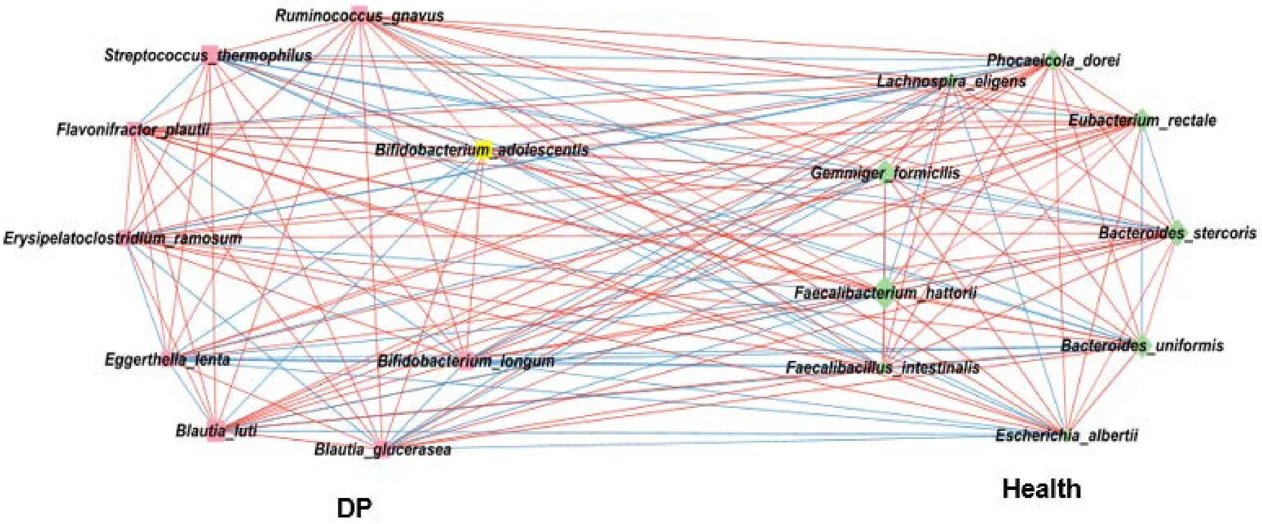

Supplement: Supplementary file 1 [file ijms-24-13329-s001.zip › Supplementary Table and figues.pdf]
